# Myogenin Regulates Exercise Capacity and Skeletal Muscle Metabolism in the Adult Mouse

**DOI:** 10.1371/journal.pone.0013535

**Published:** 2010-10-22

**Authors:** Jesse M. Flynn, Eric Meadows, Marta Fiorotto, William H. Klein

**Affiliations:** 1 Department of Biochemistry and Molecular Biology, The University of Texas M. D. Anderson Cancer Center, Houston, Texas, United States of America; 2 Graduate Training Program in Genes and Development, The University of Texas School of Biomedical Sciences at Houston, Houston, Texas, United States of America; 3 Department of Pediatrics, United States Department of Agriculture (USDA)/Agricultural Research Service (ARS) Children's Nutrition Research Center, Baylor College of Medicine, Houston, Texas, United States of America; University Hospital Vall d'Hebron, Spain

## Abstract

Although skeletal muscle metabolism is a well-studied physiological process, little is known about how it is regulated at the transcriptional level. The myogenic transcription factor myogenin is required for skeletal muscle development during embryonic and fetal life, but myogenin's role in adult skeletal muscle is unclear. We sought to determine myogenin's function in adult muscle metabolism. A *Myog* conditional allele and Cre-ER transgene were used to delete *Myog* in adult mice. Mice were analyzed for exercise capacity by involuntary treadmill running. To assess oxidative and glycolytic metabolism, we performed indirect calorimetry, monitored blood glucose and lactate levels, and performed histochemical analyses on muscle fibers. Surprisingly, we found that *Myog*-deleted mice performed significantly better than controls in high- and low-intensity treadmill running. This enhanced exercise capacity was due to more efficient oxidative metabolism during low- and high-intensity exercise and more efficient glycolytic metabolism during high-intensity exercise. Furthermore, *Myog*-deleted mice had an enhanced response to long-term voluntary exercise training on running wheels. We identified several candidate genes whose expression was altered in exercise-stressed muscle of mice lacking myogenin. The results suggest that myogenin plays a critical role as a high-level transcriptional regulator to control the energy balance between aerobic and anaerobic metabolism in adult skeletal muscle.

## Introduction

In all vertebrate organisms, including humans, skeletal muscle is composed of a variety of muscle fiber types, each with specialized roles that support the body's skeleton and enable the organism to perform a wide range of movements [1]. Underlying each of these distinct roles are numerous genes whose expression must be tightly controlled to achieve the desired muscle response. Studies on the control of muscle fiber type gene expression have revealed the signaling pathways and transcriptional regulators that are critical for ensuring that the correct balance of fiber types fits the needs of the organism. Despite this knowledge, major questions still remain as to how myofiber types are in fact coupled to muscle metabolism and whether genes associated with metabolism and energy utilization are regulated by transcriptional mechanisms separate from those that define fiber types.

In nonhuman adult mammals, myofibers are classified by the specific expression of myosin heavy-chain (MHC) isoforms into type I, type IIa, IIx/d and type IIb fibers [2]. Fiber types I and IIa exhibit oxidative metabolism, and type IIX/d and IIb are primarily glycolytic [3]. Type I myofibers, also called slow twitch fibers, exert slow contractions due to the ATPase activity associated with type I fibers [4]. Type I and type IIa fibers are rich in mitochondria and have more capillaries surrounding them than do type IIx/d and IIb fibers [4]. Type I and type IIa fibers also exhibit a high resistance to fatigue. In contrast, type IIb muscle fibers, also called fast twitch fibers, exert quick contractions and rapidly fatigue. Thus, slow oxidative muscle fibers are necessary for skeletal support and endurance, while fast glycolytic fibers are used when the organism requires movements involving strength and speed [1]. The mechanisms involved in remodeling muscle fiber types are distinct from the mechanisms used by satellite cells (adult muscle stem cells) to regenerate skeletal muscle. In fact, myofiber remodeling appears to be under the control of separate signaling pathways than those required for satellite cell activation [5].

The four myogenic basic helix-loop-helix regulatory factors, MyoD, Myf5, myogenin, and MRF4, are crucial for muscle development in the embryo. We were interested in the possibility that one or more of these factors might play additional roles in muscle metabolism and energy utilization in adult life. Myogenin is best known for regulating skeletal muscle development during the embryonic and fetal stages of life [6]. Myogenin is required for embryonic myoblast differentiation, and *Myog*-null mice die at birth due to severe skeletal muscle deficiency [7]. In contrast to its role in prenatal life, a function for myogenin during adult life is less certain. Expression of *Myog* persists in adult myofibers, albeit at reduced levels, suggesting that myogenin plays a role in skeletal muscle maintenance and repair. We recently generated a mouse line harboring a conditional *Myog* allele (*Myog^flox^*) and a Cre-ER transgene that allows us to investigate the role of myogenin in the adult mouse [8]. When *Myog* is deleted shortly after birth (P1), skeletal muscle growth is undisturbed and all adult skeletal muscle groups are seemingly normal despite the fact that mice increase their muscle mass by more than 20-fold from birth to maturity [9]. However, P1 *Myog*-deleted mice are approximately 30% smaller in size than wild-type littermates, suggesting that the loss of myogenin affects overall body homeostasis. Because of myogenin's importance during embryonic and fetal life, the results with P1 *Myog*-deleted mice were unexpected. Nonetheless, they leave open the possibility that if *Myog*-deleted adult mice were placed under conditions that required more than routine muscle stress, a role for myogenin might become apparent.

Recent studies have suggested a possible link between myogenin and adult myofiber maintenance and survival. Tang et al. (2009) and Moresi et al. (2010) have demonstrated that *Myog*-deleted mice are resistant to muscle atrophy following denervation. In muscle atrophy, myogenin was shown to function as an activator of ubiquitin ligases, which are required for proteosome-mediated protein degradation [10]. *Myog* mRNA is preferentially expressed in adult slow-twitch muscle fibers, whereas *MyoD* transcripts are of greater abundance in fast-twitch fibers [11]. In cell culture, low doses of myogenin promote the formation of fast-twitch fibers [12]. Furthermore, myogenin has been shown to induce a shift in enzyme activity from glycolytic to oxidative metabolism when overexpressed in adult mice [13]. These studies set the stage for examining the regulatory roles that myogenin might play in muscle metabolism and energy utilization.

In this current study, we show that *Myog*-deleted adult mice exhibit an enhanced exercise capacity in response to physical stress and are able to more fully deplete their glycogen stores before reaching exhaustion compared with wild-type controls. In addition, *Myog*-deleted mice display an enhanced response to exercise training when given long-term access to running wheels. Gene expression analysis with muscle from *Myog*-deleted mice reveals alterations in the expression levels of transcripts involved in aerobic and anaerobic metabolism. Our results indicate that myogenin serves as a high-level transcription factor to regulate muscle metabolism and energy utilization.

## Results

### Myog-deletion has minor effects on sedentary adult mice

Administration of tamoxifen by intraperitoneal injection of P1 pups effectively deletes the floxed *Myog* allele but results in significantly smaller adult mice [9]. We were concerned that using mice weighing less than wild-type controls would complicate our exercise performance experiments. Accordingly, we developed a simple procedure to delete *Myog* in mature adults by intraperitoneal injection of tamoxifen. Intraperitoneal injection successfully led to an 85–99% deletion of *Myog* from tail and gastrocnemius skeletal muscle DNA (Fig. S1A). RT-qPCR analysis revealed a substantial reduction in *Myog* transcripts in Myog-deleted mice (Fig. S1B). For our experiments, we used mice injected between 8–12 weeks of age with greater than 90% *Myog* deletion. For all experiments, the wild-type control group consisted of tamoxifen-treated mice homozygous for *Myog* ^flox^ without Cre-recombinase. The effects of myogenin deletion on body mass, composition, and metabolic parameters on sedentary male mice at one year of age are summarized in Table 1. No changes in body mass were observed within three months after *Myog* deletion (Fig. S1C), but after one year, *Myog*-deleted mice weighed 9.4% less than wild-type controls, with the difference in weight distributed proportionally between lean and fat. No differences in food consumption were detected at this one-year time point and there were also no differences in average 24 hr energy expenditure, resting metabolic rate or respiratory exchange ratio (RER). Overall, the absence of myogenin appeared to have only subtle effects in the unstressed, sedentary situation.

**Table 1:** Comparison of body composition and metabolic parameters in 1-year-old sedentary *Myog*-deleted and wild-type control mice.

	*Myog* -Deleted	Wild-type	P
N	8	6	
Body weight (g)	31.6±1.5	34.1±2.5	0.04
Lean Mass (g)	24.5±1.3	26.6±2.2	0.05
Fat Mass (% body weight)	8.3±2.7	8.3±0.9	NS
24-h Food Intake (g/d)*	3.71±0.13	3.84±0.11	NS
24-h Energy Expenditure (kcal/h) '	0.45±0.01	0.45±0.01	NS
Resting Metabolic Rate (kcal/h) '	0.32±0.01	0.30±0.01	NS
24-h Average RER	0.90±0.05	0.88±0.05	NS
12-h Light average RER	0.85±0.04	0.83±0.03	NS
12-h Dark average RER	0.96±0.03	0.94±0.03	NS
24-h Activity (total counts)	325923±5573	26703±2920	0.04
12-h Light activity (total counts)	8234±1549	6709±1613	NS
12-h Dark activity (total counts)	24348±4431	19994±3126	0.06

Values are means ± SD.

*Values are Least Square Means that have been normalized body weight;

'Values are Least Square Means that have been normalized for lean mass. Activity counts represent the total number of beam breaks in both the X and Z dimensions. RER = respiratory exchange ratio.

### Myog-deleted adult mice exhibit an enhanced capacity for exercise

**Figure 1:**
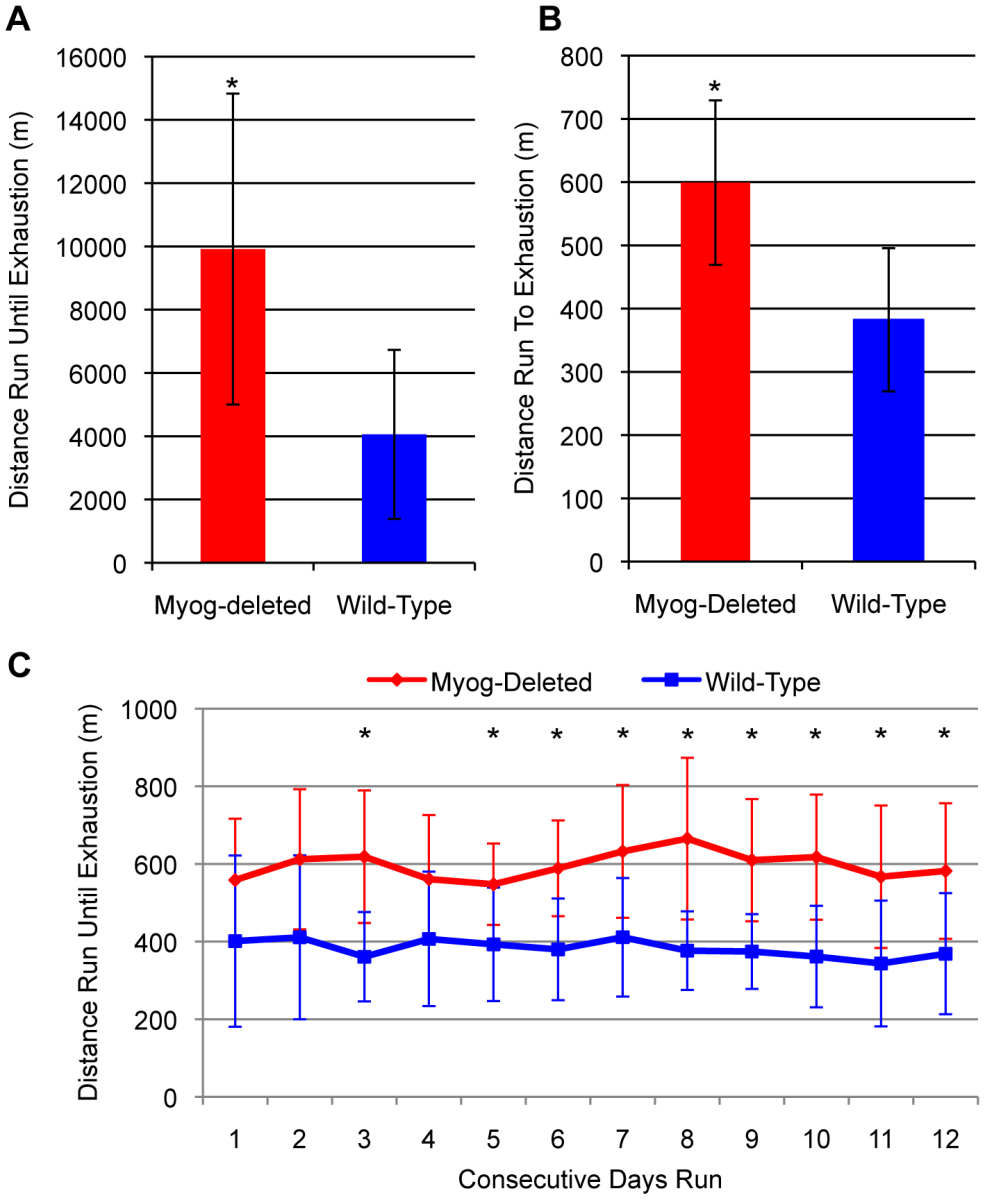
Enhanced exercise capacity in low- and high-intensity running regimens in *Myog*-deleted mice. (A) Low intensity exercise. During low-intensity exercise, *Myog*-deleted mice ran 2.4-fold farther than did wild-type mice (*Myog*-deleted, n = 4; wild-type n = 6). Blue bar indicates wild-type control values; red bar indicates *Myog*-deleted values. Error bars represent one standard deviation (*P<*0.05). (B, C) High-intensity exercise. During 12 consecutive days of high-intensity exercise, *Myog*-deleted mice ran 1.6-fold farther than did wild-type mice on average (wild-type, n = 7; *Myog*-deleted, n = 8). Blue line (B) and blue bar (C) indicate wild-type control values; red line (B) and red bar (C) indicate *Myog*-deleted values. Error bars represent one standard deviation. *P<0.01.

To place stress on skeletal muscle and monitor physical performance, we used involuntary (forced) running on a treadmill and determined exercise capacity under high- and low-intensity running regimens (Fig. S2). We used low-intensity running at 20 m/minute to assay for muscle endurance and oxidative metabolic capacity. The *Myog*-deleted mice statistically outperformed their littermate controls, showing an overall difference of 144% in the distance run (Fig. 1A). On average, wild-type mice ran 4059 m until exhaustion, whereas *Myog*-deleted mice ran 9916 m. These results were unanticipated, because we expected the absence of myogenin to negatively affect endurance exercise performance. We then subjected mice to a high-intensity protocol that increased average speed stepwise every 2 minutes until exhaustion (Fig. S2). On average, wild-type mice ran 382 m daily until exhaustion, whereas *Myog*-deleted mice ran 596 m daily until exhaustion (Fig. 1C). This response was reproducible over 12 days during which *Myog*-deleted mice ran 56% further than did control mice (Fig. 1B). Myogenin's role in adult skeletal muscle therefore seemed to be different from its role in embryonic and fetal muscle. The enhanced exercise capacity of *Myog*-deleted mice during both high- and low-intensity running supported the hypothesis that myogenin functions in modulating normal skeletal muscle metabolic activity that had not been detected in previous studies.

To determine how quickly the *Myog*-deleted exercise performance phenotype manifested after deletion of *Myog*, a large cohort of young adult mice were subjected to high-intensity treadmill running within 10 days of tamoxifen injection. On average, *Myog*-deleted mice ran 36% further than wild-type mice (547 m compared with 402 m; Fig. S3A). In these experiments, we observed modest differences in exercise capacity between *Myog*-deleted males and *Myog*-deleted females, but both sexes consistently outperformed wild-type mice of the same sex (Fig. S3B, S3C). The results demonstrated that enhanced exercise capacity was rapidly established in *Myog*-deleted mice and suggested that the responsible changes in skeletal muscle gene expression were likely to have occurred very soon following the disappearance of myogenin.

As the absence of myogenin appeared to confer an enhanced capacity for exercise, we sought to determine the normal function and expression level of *Myog* in wild-type mice before and after exercise. After running to exhaustion, on average, there was no difference in *Myog* transcript abundance in mice immediately after high- or low-intensity exercise when compared to unexercised mice (Fig. S4A). However, we found a significant correlation between the distance run during low-intensity exercise and the level of *Myog* expressed immediately after low-intensity exercise (Fig. S4B). Mice that ran the least displayed the lowest levels of *Myog* whereas mice that ran the most exhibited the highest levels of *Myog*. We did not observe this correlation during high-intensity exercise. These data suggest that *Myog* expression is normally low in adult muscle, but it is upregulated after long periods of exercise in proportion to the amount of low-intensity running performed.

### The absence of myogenin alters metabolite concentrations during exhaustive exercise

A useful way to address the basis for which *Myog*-deleted mice outperformed wild-type controls was to measure lactate and glucose levels in the bloodstream as indicators of glycolytic and oxidative capacity. During low-intensity exercise, lactate produced by glycolytic skeletal muscle enters into the interstitium and bloodstream, and is largely oxidized by the more oxidative skeletal muscle fibers and other tissues, such as the heart [14]. Some lactate, via the lactate shuttle or Cori cycle, serves as a precursor for glucose production via gluconeogenesis [15]. Glucose is then released into the circulation and is available to skeletal muscle for further glycolysis. A build-up of blood lactate would be expected after running until exhaustion under high-intensity exercise conditions and is indicative of glycolytic metabolism and the inability of the body to use the excess lactate that is produced. In contrast, unchanged blood lactate levels would be expected under low-intensity exercise conditions and are indicative of oxidative metabolism and the ability of the body to use any lactate that is produced [16]. We determined blood lactate and blood glucose levels in *Myog*-deleted mice and wild-type controls. For both genotypes, we measured blood levels in pre-exercise mice and after mice were run to exhaustion under low- and high-intensity treadmill exercise.

**Figure 2:**
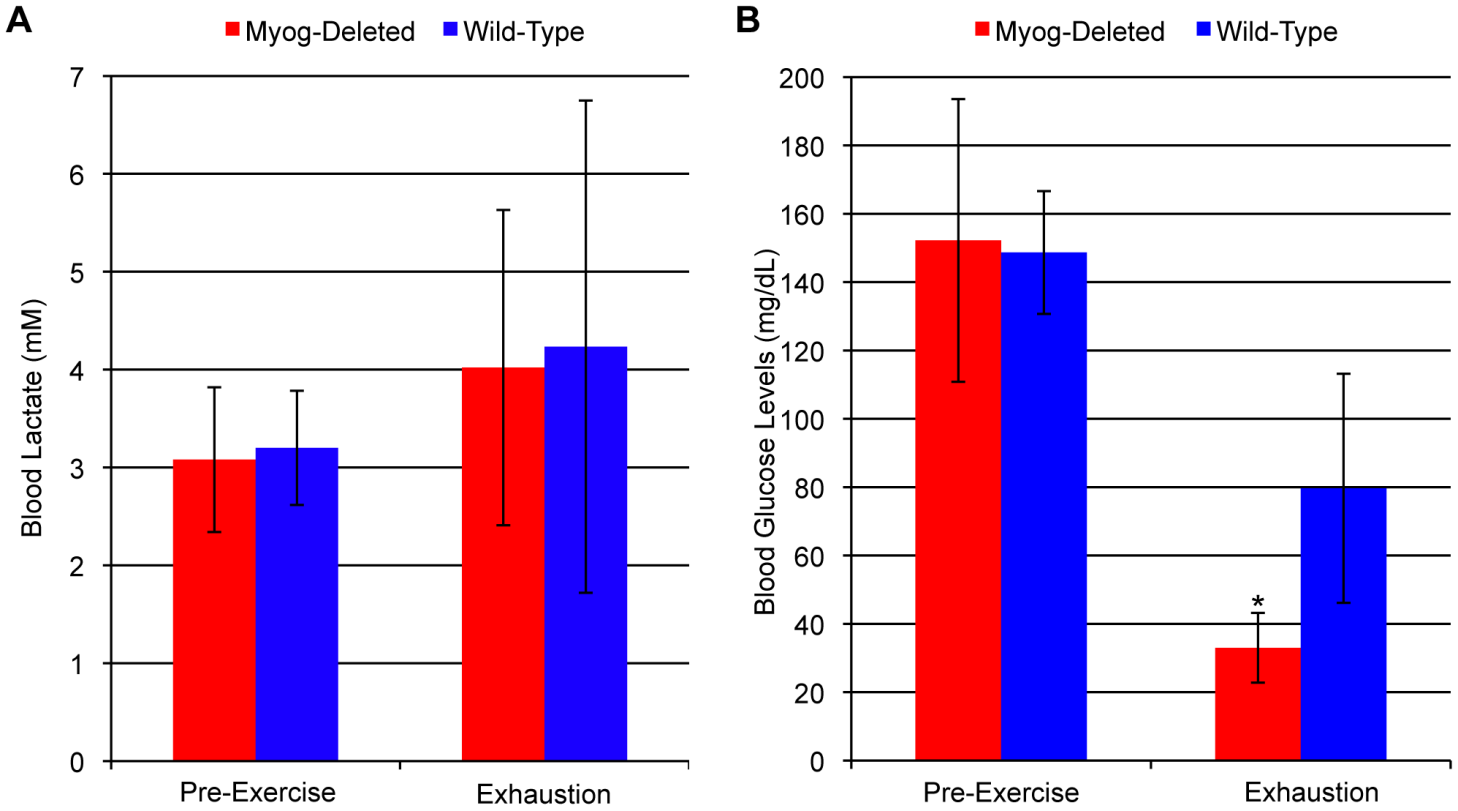
Blood metabolite values in wild-type and *Myog*-deleted mice after low-intensity exercise. (A) Blood lactate levels. Blood lactate levels in *Myog*-deleted mice were similar to those for wild-type mice before and after low-intensity exercise (Myog-deleted, 4.2 mM; wild-type, 4.5 mM). Blue bars indicate wild-type control values; red bars indicate *Myog*-deleted values. Error bars represent one standard deviation. (B) Blood glucose levels. *Myog*-deleted mice have further reduced blood glucose levels after low-intensity running. Blood glucose levels in *Myog*-deleted mice are similar to those for wild-type mice prior to low-intensity exercise (149 mg/dL). After low-intensity exercise exhaustion, blood glucose levels were reduced 78% in *Myog*-deleted mice compared with 46% for wild-type controls (*Myog*-deleted, 33 mg/dL; wild-type, 80 mg/dL). Blue bars indicate wild-type control values; red bars indicate *Myog*-deleted values. Error bars represent one standard deviation. (*Myog*-deleted, n = 6; wild-type, n = 6) *P<0.05.

In both *Myog*-deleted mice and controls, blood lactate concentrations showed no significant change after running to exhaustion at low-intensity, (Fig. 2A). These results were consistent with low intensity exercise and normal oxidative metabolism. After low-intensity running, glucose levels were lower in both genotypes compared with pre-exercise glucose levels, as would be expected, but *Myog*-deleted mice had significantly lower blood glucose concentrations than did wild-type mice (Fig. 2B). The results indicated that glucose was being used preferentially in *Myog*-deleted mice during low-intensity exercise and suggested that *Myog*-deleted mice were inherently more efficient in utilizing oxidative metabolism than were wild-type mice.

**Figure 3:**
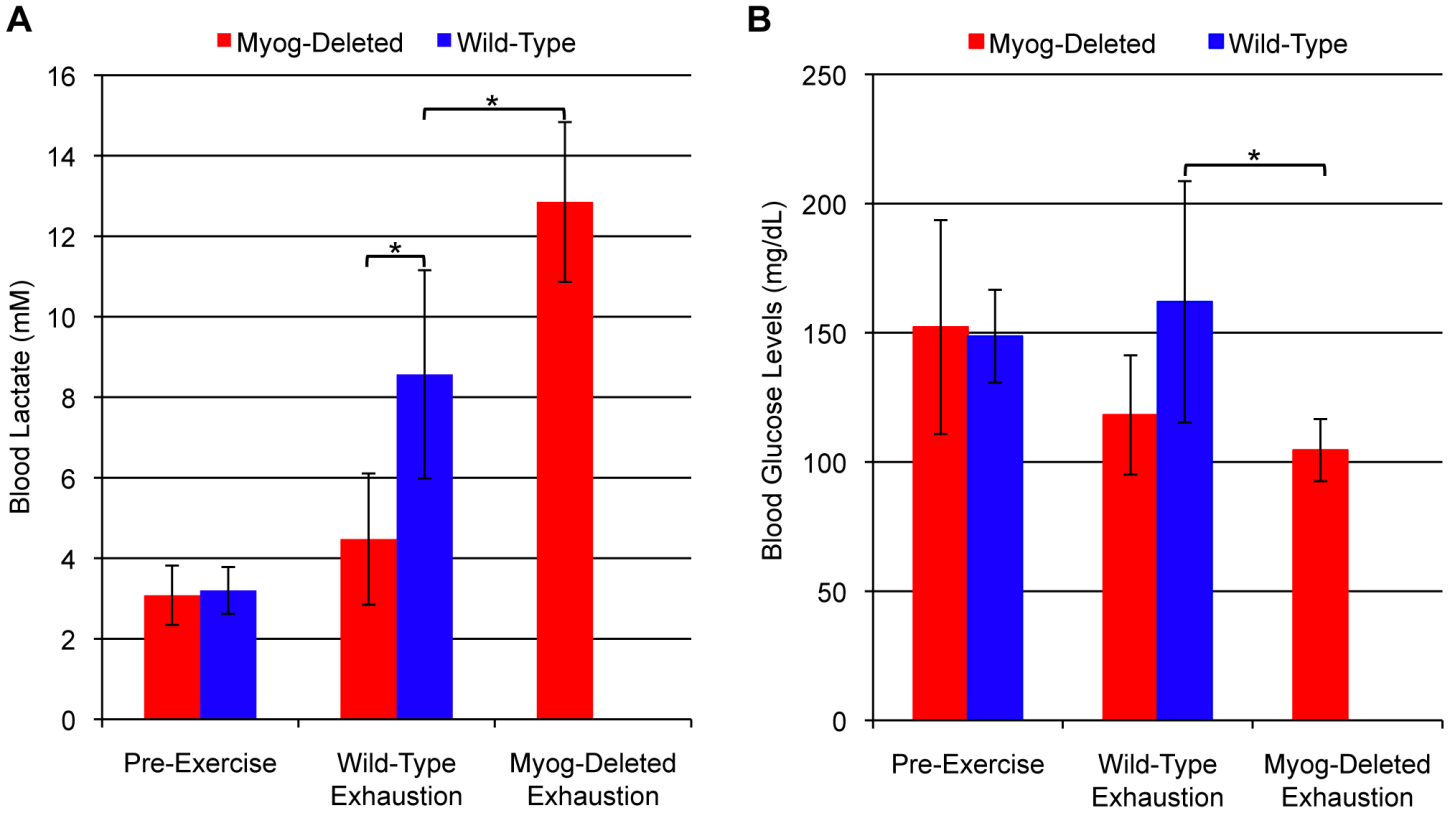
Blood metabolite values in wild-type and *Myog*-deleted mice after high-intensity exercise. (A) Blood lactate levels. Prior to exercise, *Myog*-deleted mice exhibited normal levels of blood lactate (*Myog*-deleted, 3.1 mM; wild-type, 3.2 mM). Exhausted wild-type mice exhibited elevated blood lactate levels (8.6 mM), 2.7-fold higher than pre-exercise levels. At wild-type exhaustion, *Myog*-deleted mice had blood lactate levels similar to pre-exercise levels. Exhausted *Myog*-deleted mice exhibited a 4.1-fold increase in blood lactate relative to pre-exercise levels and 1.5-fold higher than exhausted wild-type mice (12.9 mM) (*Myog*-deleted, n = 6; wild-type, n = 6). Blue bars indicate wild-type control values; red bars indicate *Myog*-deleted values. Error bars represent one standard deviation (*P<*0.05). (B) Blood glucose values. Prior to exercise, *Myog*-deleted mice had normal blood glucose levels compared with wild-type control mice (52 mg/dL). When wild-type mice reached exhaustion, their blood glucose levels were similar to their pre-exercise values (162 mg/dL). At exhaustion, *Myog*-deleted mice had a 33% reduction in blood glucose relative to levels in wild-type mice at exhaustion (*Myog*-deleted, n = 6; wild-type, n = 6). Blue bars indicate wild-type control values; red bars indicate *Myog*-deleted values. Error bars represent one standard deviation. *P<0.05.

Another group of mice was subjected to high-intensity running. When wild-type mice reached exhaustion, their blood lactate levels were elevated 2.7 fold compared with the basal levels of pre-exercised mice whereas after the same amount of exercise, blood lactate levels in the *Myog-*deleted mice were not different from baseline (Fig. 3A). However, when *Myog*-deleted mice were run to exhaustion, there blood lactate was elevated 4 fold (Fig. 3A). These highly elevated blood lactate levels were a strong indication of enhanced glycolysis and lactate production in the skeletal muscles of *Myog*-deleted mice that had been run to exhaustion. Furthermore, at exhaustion *Myog*-deleted mice had reduced blood glucose levels compared to the wild-type mice (Fig. 3B). Reduced blood glucose was another strong indicator that metabolism processes were enhanced during high-intensity exercise in Myog-deleted mice.

Increased muscle and liver glycogen stores may be responsible for the enhanced exercise capacity of *Myog*-deleted mice. We did not detect any significant differences in total soleus muscle or liver glycogen content in *Myog*-deleted mice when compared to wild-type mice (Fig. S5); total glycogen content in EDL muscle was also unchanged (data not shown). However, under the high-intensity exercise regimen, *Myog*-deleted mice significantly depleted both their liver and skeletal muscle (soleus) glycogen reserves by 63% and 51%, respectively, whereas the decreases seen in wild-type liver and muscle glycogen content were milder and not statistically significant, 51% and 27%, respectively. These results indicate that *Myog*-deleted mice have similar liver and muscle glycogen content at rest, but are capable of more extensively depleting their glycogen stores to continuously fuel their metabolisms. The excessive production of lactate and increased utilization of muscle glycogen as a source of fuel suggested that *Myog*-deleted mice exhibited an increased glycolytic flux during high-intensity exercise. Virtually unchanged *Myog*-deleted lactate levels at the wild-type exhaustion point and increased utilization of liver glycogen before reaching exhaustion suggested that *Myog*-deleted mice possessed enhanced oxidative capacity.

### *Myog*-deleted mice employ oxidative metabolism longer while running before reaching exhaustion

To further address the metabolic changes occurring in the absence of myogenin, wild-type and *Myog*-deleted mice were run under the high-intensity regimen while measuring their oxygen consumption and carbon dioxide production. In both groups of mice, after the initial changes at the start of the exercise period, the average RER remained fairly constant and did not differ between groups (0.925±0.029 and 0.924±0.034, in *Myog*-deleted and wild-type, mice respectively). As the mice approached exhaustion, there was a sharp upward inflection in both VCO_2_ and VO_2_ with the increase in VCO_2_ being greater than for VO_2_. This point of inflection correlates with the lactate threshold when lactate accumulates in the blood, and resulted in RER values rising above 1.0 [17].

**Figure 4:**
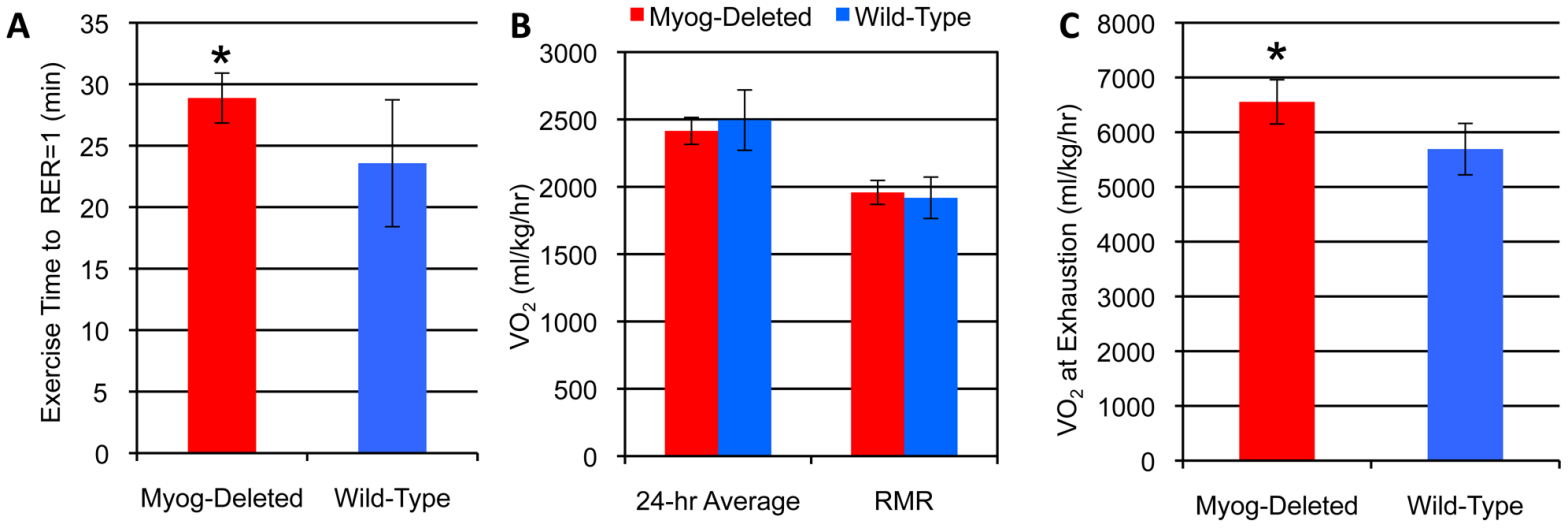
Oxygen consumption and exercise time to RER = 1 in wild-type and *Myog*-deleted mice. (A) Exercise time to RER = 1. *Myog*-deleted mice ran 22% longer than the wild-type group before transitioning from primarily oxidative metabolism to glycolytic metabolism at RER = 1. (B) VO_2_ at rest. There was no difference in sedentary 24 hr and resting metabolic rate (RMR) VO_2_ between *Myog*-deleted and wild-type controls. (C) Peak VO_2_ at exhaustion. *Myog*-deleted mice displayed 15% greater oxygen consumption at exhaustion over the wild-type group. *Myog*-deleted, n = 7; wild-type, n = 8. Blue bars indicate wild-type control values; red bars indicate *Myog*-deleted values. Error bars represent one standard deviation. *P<0.05.

*Myog*-deleted mice ran 23% longer before reaching an RER of 1.0, indicating that these mice were able to utilize oxidative metabolism for a longer period of time than their wild-type counterparts (Fig. 4A). Although under sedentary and resting conditions oxygen consumption was not different among the two groups, at exhaustion, VO_2_ values were 15% greater in *Myog*-deleted mice (Fig. 4B, 4C). This result indicates that in the absence of myogenin, *Myog*-deleted mice exhibit increased aerobic capacity during exercise, although not during inactivity. These results provide insight into how *Myog*-deleted animals are able to display an increased capacity for exercise over their wild-type counterparts.

The increase in the oxygen consumption of *Myog*-deleted mice suggests that they possess increased levels of mitochondria. We determined mitochondrial DNA copy number in gastrocnemius muscle tissue by qPCR and observed a moderate trend towards an increased ratio of mtDNA:nDNA in *Myog*-deleted mice relative to wild-type mice (Fig. S6). Thus, while *Myog*-deleted animals are likely to have increased numbers of mitochondria, this was not definitively shown to be the case because of our necessarily small sample size.

### Myogenin is not required for maintaining muscle fiber type, aerobic metabolic potential, metabolic enzyme activity, or expression of selected genes involved in metabolism

**Figure 5:**
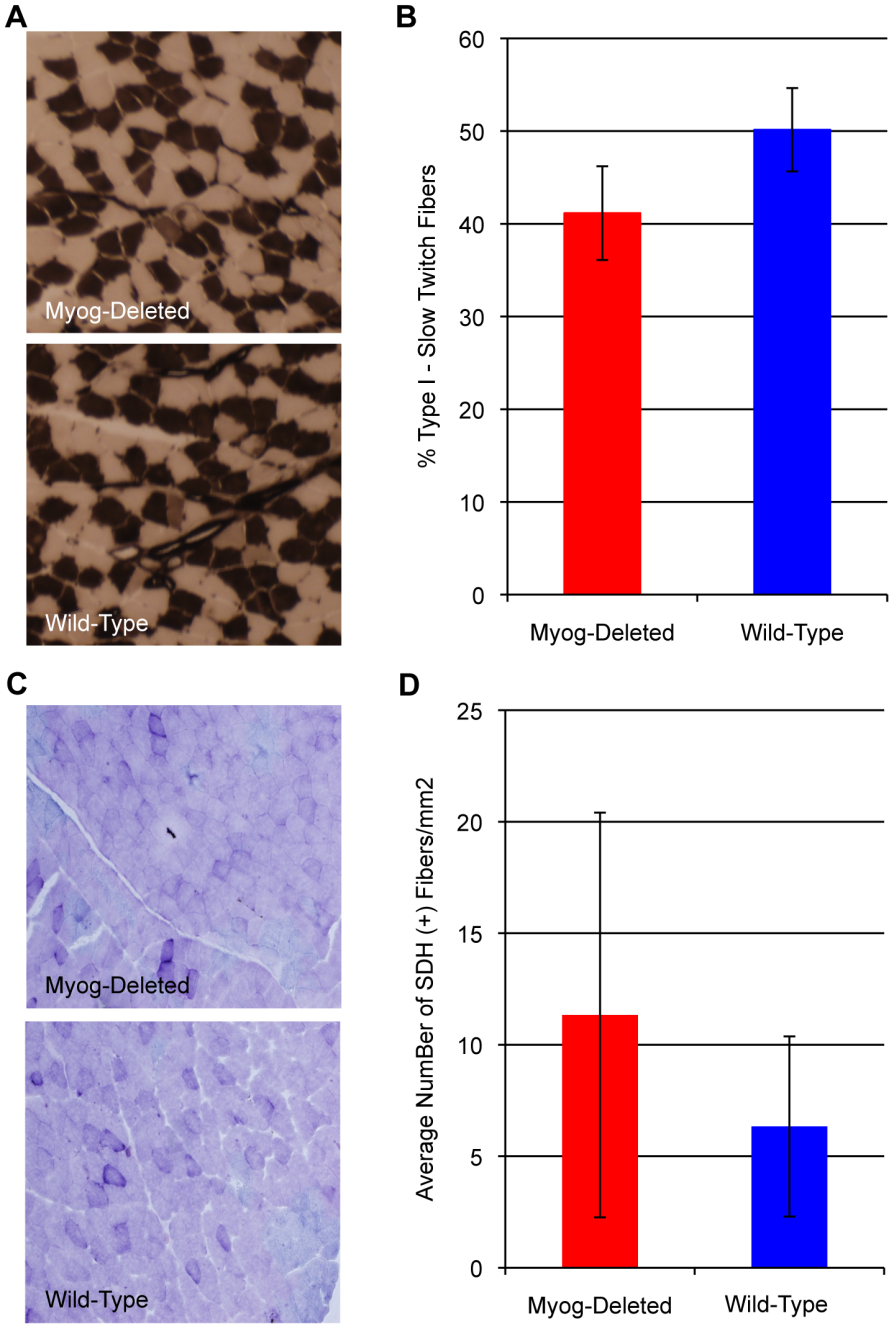
Fiber type proportion and oxidative activity in untrained wild-type and *Myog*-deleted mice. (A, B) Fiber type proportion. *Myog*-deleted mice had normal proportions of type I and type II fibers in soleus muscle. Myosin ATPase staining was used to identify type I and type II fibers in the soleus muscle (A). Average quantified values revealed normal proportions of fiber types (B). Blue bar indicates wild-type control values; red bar indicates *Myog*-deleted values. Error bars represent one standard deviation. (C, D) Oxidative activity. *Myog*-deleted mice had no difference in SDH activity in gastrocnemius. SDH staining was used to identify oxidative fibers in the medial gastrocnemius (C). Average quantified values revealed normal oxidative activity (D). Blue bar indicates wild-type control values; red bar indicates *Myog*-deleted values. *Myog*-deleted, n = 3; wild-type, n = 3. Values for each mouse were averaged from the analyses of two 1 mm^2^ fields. Error bars represent one standard deviation.

The experiments that we performed in the low- and high-intensity exercise regimens implied a greater reliance on oxidative metabolism in *Myog*-deleted mice. The observed enhancement of exercise performance could have resulted from an increase in the proportion of slow twitch fibers and a corresponding increase in mitochondrial citric acid cycle activity. We used histochemical staining procedures for type I and type II myosin-ATPases and succinate dehydrogenase (SDH) activity to determine whether fiber type switching or enhanced citric acid cycle activity occurred in skeletal muscles of *Myog*-deleted mice. SDH activity is an indicator of overall aerobic potential. We found no difference between *Myog*-deleted mice and wild-type controls in the proportion of type I and type II myofibers in soleus or gastrocnemius muscle, as determined by myosin-ATPase staining (Fig. 5A, 5B). Furthermore, myosin type-I, type-IIa, and type-IIb isoform transcript expression levels were unchanged in gastrocnemius muscle (data not shown). SDH activity revealed no differences in the proportion of oxidative myofibers in gastrocnemius muscle in *Myog*-deleted mice compared with wild-type controls (Fig. 5C, 5D). We also determined the expression levels of eight genes encoding proteins that function in fatty acid metabolism and mitochondrial function, in addition to glucose transporter 4 (Glut4), and found an increase in cytochrome c oxidase 8b (Cox8b) and peroxisome proliferator-activated receptor gamma (Ppar-g) transcripts; and a decrease in Glut4 and Uncoupling Protein 3 (UCP3) transcripts (Fig. S7). Furthermore, the activity levels of enzymes regulating both glycolytic and oxidative metabolic processes did not reveal differences (Fig. S8). The results indicated that many adaptations that traditionally accompany enhanced exercise endurance were not found in *Myog*-deleted mice.

### Hypoglycemia abolishes enhanced exercise capacity in Myog-deleted mice

Enhanced exercise capacity in *Myog*-deleted mice could be associated with increased carbohydrate consumption and replenishment of glycogen stores. During a 5-day period of treadmill running, *Myog*-deleted mice consumed normal amounts of food (data not shown). The results suggested that *Myog*-deleted mice did not require increased dietary consumption to maintain their enhanced exercise capacity.

**Figure 6:**
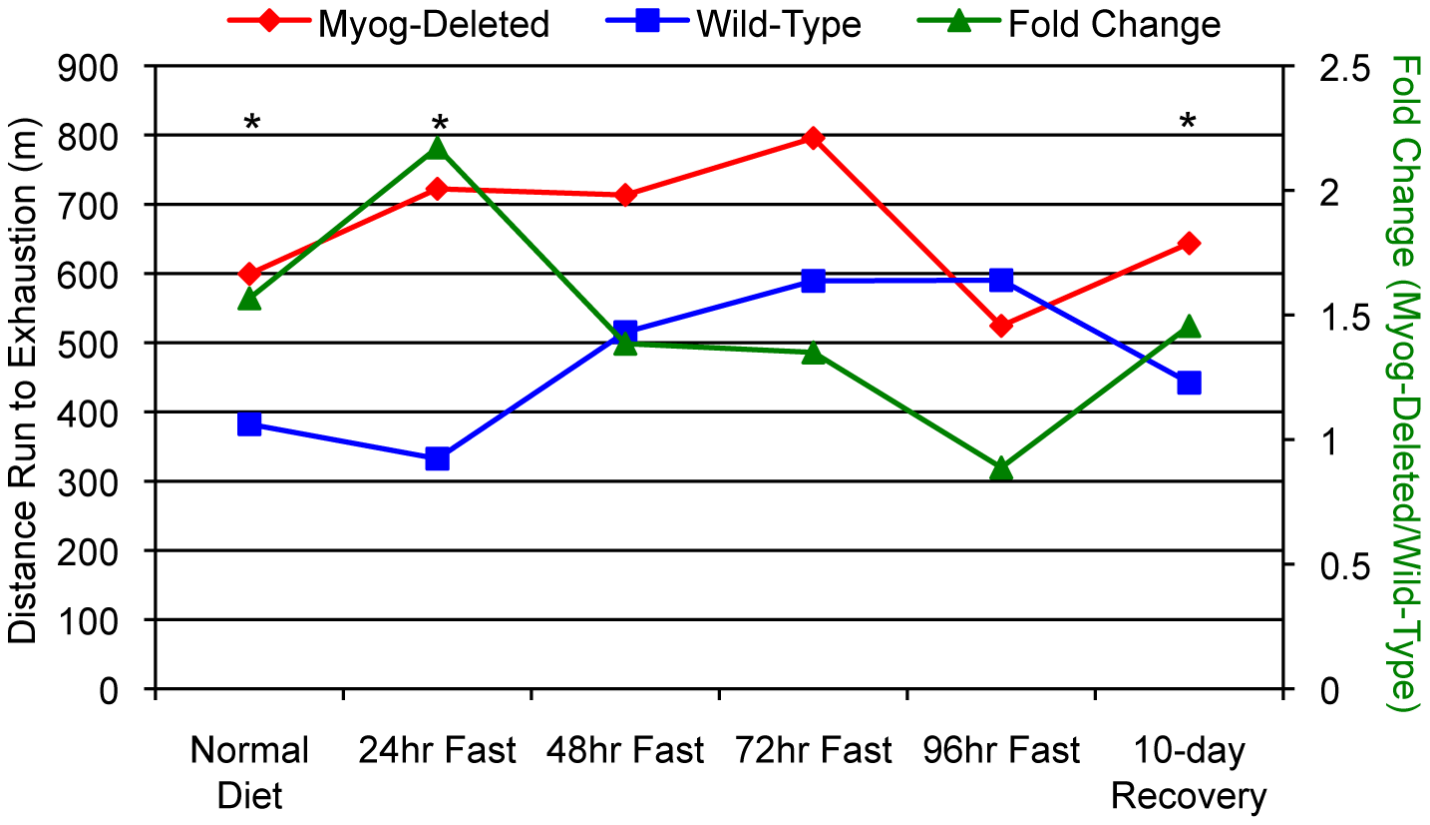
High intensity running in hypoglycemic wild-type and *Myog*-deleted mice. Mice were subjected to high-intensity exercise before, during, and after 96 hours of food deprivation. Under normal dietary conditions, *Myog*-deleted mice ran approximately 1.6-fold farther than did wild-type control mice. After 24 hours of food deprivation, *Myog*-deleted animals ran 2-fold farther than did wild-type control mice. At 96 hours of food deprivation, *Myog*-deleted mice lost their enhanced exercise endurance. Upon refeeding, the enhanced exercise endurance of *Myog*-deleted mice was restored within 10 days. The daily increase in running distance between 24 and 72 hours was concomitant with a daily loss of 10% body mass (not shown). Blue line indicates wild-type control values; red line indicates *Myog*-deleted values; green line indicates fold change of *Myog*-deleted values relative to wild-type values. *P<0.05.

To determine whether enhanced exercise capacity was dependent on a sufficient supply of glucose for glycolysis, *Myog*-deleted mice were fasted for 96 hours and exercised to exhaustion daily with the high-intensity running regimen (Fig. 6). After 24 hours of food deprivation, *Myog*-deleted mice continued to outperform wild-type control mice by 2.2 fold, but by 48, 72, and 96 hours, *Myog*-deleted mice no longer statistically outperformed wild-type mice (Fig. 6). We then placed the mice on their normal diet and determined their exercise capacity after 10 days. *Myog*-deleted mice once again significantly outperformed wild-type controls (Fig. 6). Throughout this experiment, *Myog*-deleted mice and wild-type controls had similar body weights. Blood lactate and glucose concentrations were determined before and after running to exhaustion during the periods of food deprivation. After 96 hours of fasting, pre- and post-exercise blood lactate and glucose concentrations of *Myog*-deleted mice were the same as those of wild-type mice (data not shown). The results indicated that a sufficient store of glycolytic substrates was required for enhanced exercise capacity in *Myog*-deleted mice.

### Myog-deleted mice do not voluntarily run more but are inherently adapt better to exercise training than wild-type mice

Enhanced involuntary exercise endurance might be the result of increased resistance to fatigue or a tendency for increased physical activity. Two approaches were used to determine whether *Myog*-deleted mice had a tendency for increased activity. We determined their running capacity in voluntary running, as well as their spontaneous cage activity measured using an infra-red beam monitoring procedure. Wild-type and *Myog*-deleted mice were allowed unrestricted access to running wheels. Over a period of 5 months, *Myog*-deleted mice did not run significantly farther or have higher average and maximum speeds than did wild-type mice on running wheels without a weight load (data not shown). Similar results were obtained when a 1-kg load was applied (data not shown). The lack of enhanced exercise capacity during voluntary running indicated that the absence of myogenin did not confer an increased tendency for wheel-running exercise. However, the measurement of spontaneous activity from the total number of infrared beam breaks over 24 hr in both the X and Z dimensions showed there was an 18% higher level of activity in *Myog*-deleted mice (P<0.04) with a tendency (P = 0.06) for movement in the Z-dimension (rearing) to show the greatest difference (Table 1).

**Figure 7:**
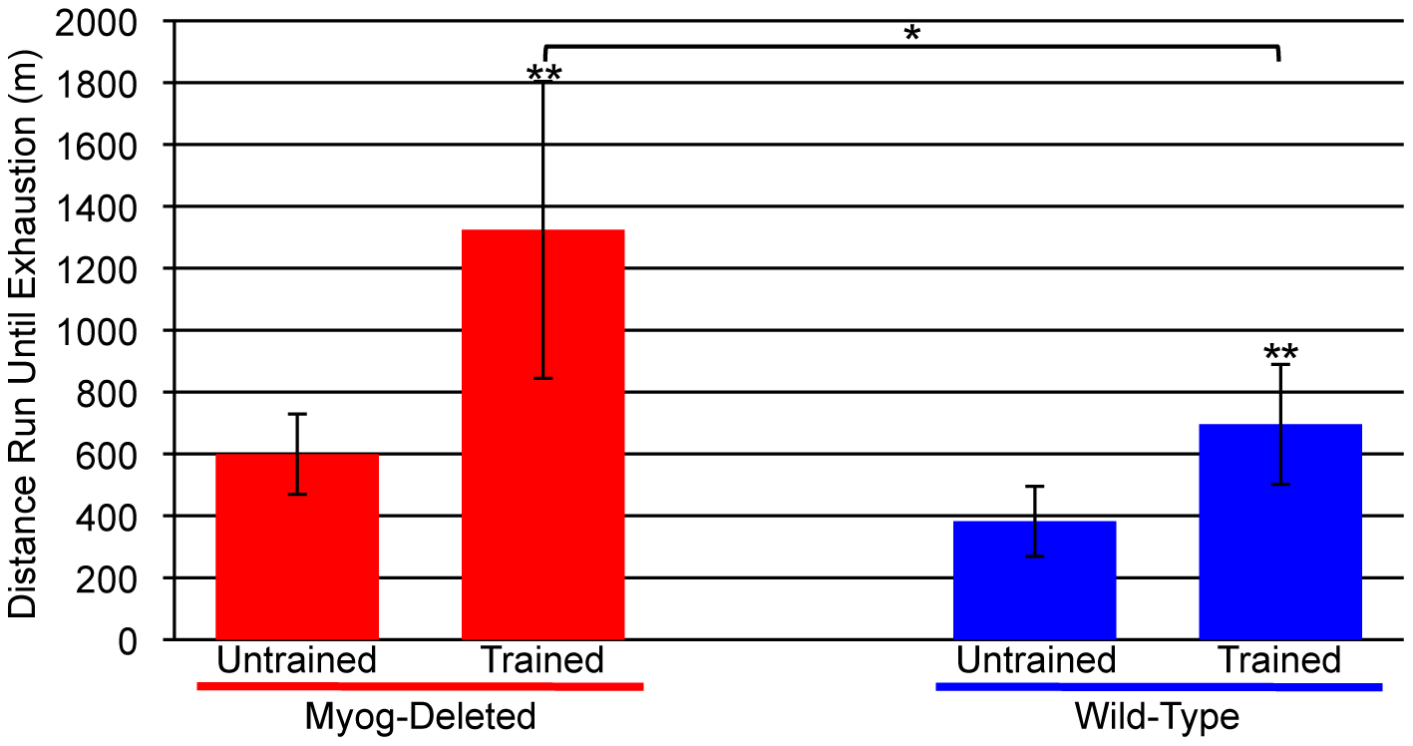
Superior adaptation to exercise training in *Myog*-deleted mice. After voluntary exercise training (trained) or during sedentary life (untrained), *Myog*-deleted and wild-type mice were subjected to at least 12 days of high-intensity treadmill running. Trained wild-type mice showed a typical adaptation to exercise training, running an average 1.8-fold more than untrained wild-type mice. Trained *Myog*-deleted mice showed a superior adaptation to exercise training, running an average 2.2-fold more than untrained *Myog*-deleted mice. Trained *Myog*-deleted mice exhibited a 20% increase in their adaptive response to training relative to trained wild-type mice (untrained wild-type, n = 7; untrained *Myog*-deleted, n = 8; trained wild-type, n = 10; trained *Myog*-deleted, n = 11). Blue bars indicate wild-type control values; red bars indicate *Myog*-deleted values. Error bars represent one standard deviation. *P<0.05, **P<0.01.

It is also possible that the absence of myogenin allowed mice to better adapt to long-term exercise-induced training. To determine whether *Myog*-deleted mice were abnormally effected by exercise training, mice that were allowed unrestricted access to voluntary running wheels for 6 months (referred to as trained mice) were subjected to high-intensity treadmill running. Wild-type mice had a significant response to training, showing a 1.8-fold increase in exercise capacity during high-intensity treadmill running (Fig. 7). *Myog*-deleted mice also exhibited a significant response to training, with a 2.2-fold increase in exercise capacity (Fig. 7). Notably, a pair-wise comparison between the wild-type and *Myog*-deleted groups indicated that *Myog*-deleted mice responded 20% better to exercise training than did wild-type mice (Fig. 7). The results supported the hypothesis that *Myog*-deleted mice were inherently more sensitive to long-term exercise-induced training adaptations.

### *Myog*-deleted mice develop further increased proportions of slow twitch fibers and SDH-positive fibers in skeletal muscle in response to long-term exercise training

**Figure 8:**
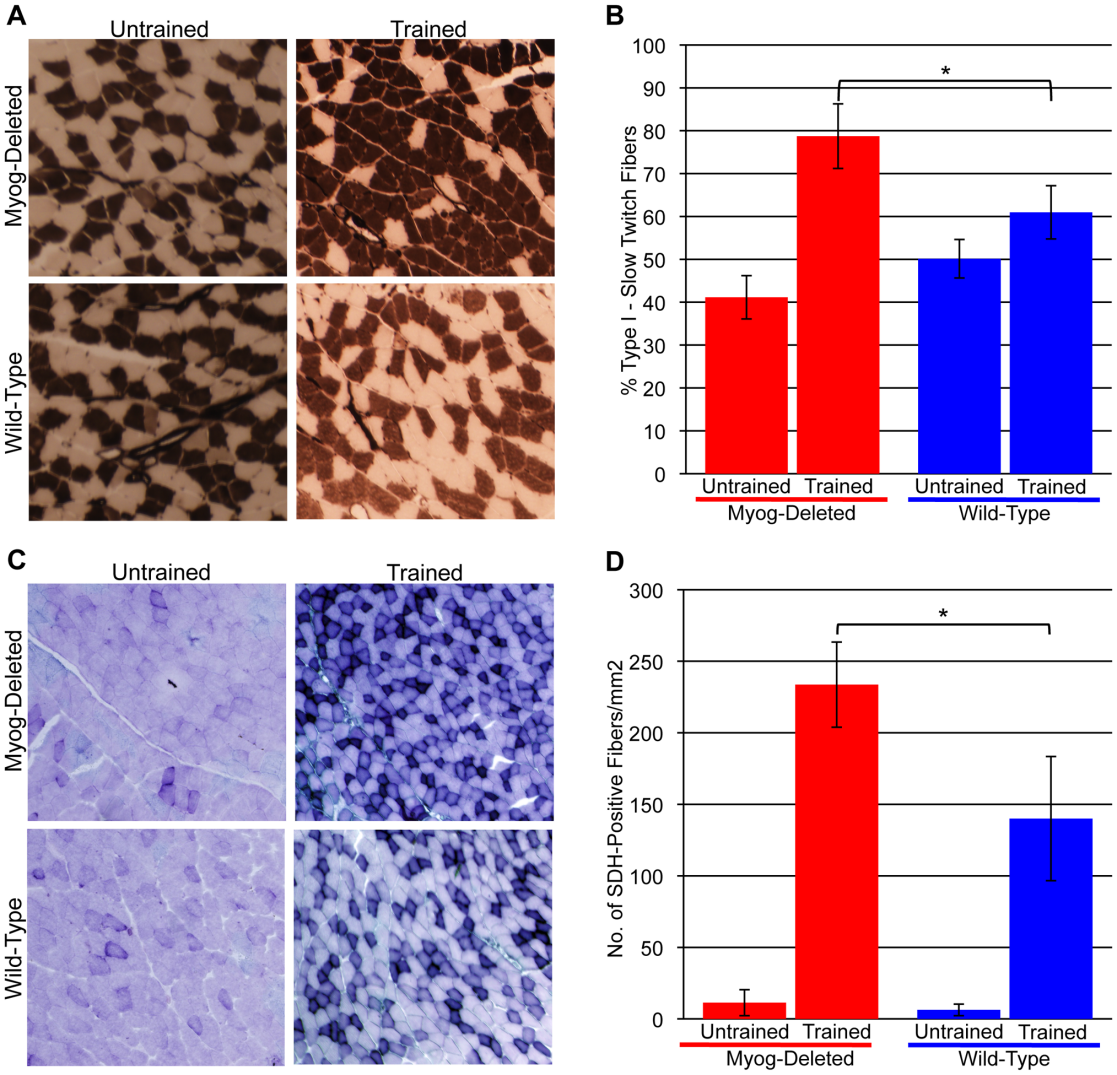
Fiber type proportion and oxidative activity in untrained and trained wild-type and *Myog*-deleted mice. (A, B) Fiber type proportion. Myosin ATPase staining was used to identify type I and type II fibers in the soleus muscle of untrained and trained mice (A). Average quantified values revealed a 22% increase in type I fibers in trained wild-type mice (untrained wild-type, 50%; trained wild-type, 61%); trained *Myog*-deleted mice exhibited a 91% increase in the proportion of type I fibers (untrained *Myog*-deleted, 41%; trained *Myog*-deleted, 79%) (B). Trained *Myog*-deleted mice exhibited a 30% increase in the proportion of type I fibers relative to trained wild-type mice. Blue bars indicate wild-type control values; red bars indicate *Myog*-deleted values. Error bars represent one standard deviation (*P<0.05). (C, D) Oxidative activity. SDH staining was used to identify oxidative fibers in the gastrocnemius of untrained and trained mice (C). Average quantified values revealed a 23-fold increase in SDH-positive fibers in trained wild-type mice (untrained wild-type, 6 SDH fibers/mm^2^; trained wild-type, 140 SDH fibers/mm^2^); trained *Myog*-deleted mice exhibited a similar 21-fold increase in SDH-positive fibers (untrained *Myog*-deleted, 11 SDH fibers/mm^2^; trained *Myog*-deleted, 234 SDH fibers/mm^2^) (D). Trained *Myog*-deleted mice exhibited a 67% increase in SDH-positive fiber density relative to trained wild-type mice. Blue bars indicate wild-type control values; red bars indicate *Myog*-deleted values. Error bars represent one standard deviation. (*Myog*-deleted, n = 3; wild-type, n = 3). Values for each mouse were averaged from the analyses of two 1 mm^2^ fields. *P<0.05.

*Myog*-deleted mice subjected to low-intensity exercise showed no significant differences in slow and fast twitch fiber type proportions or SDH activity compared with wild-type control mice (see Fig. 5). However, the possibility existed that mice lacking myogenin were inherently prone to fiber type switching and that histological changes in myofibers might be uncovered under conditions promoting long-term endurance. To test this possibility, we determined myosin-ATPase and SDH activities in histological sections from our group of trained *Myog*-deleted and wild-type mice. Myosin-ATPase staining for fast and slow twitch fibers showed that training modestly increased the proportion of type I slow-twitch fibers in the soleus muscle of wild-type mice (Fig. 8A, 8B). Trained *Myog*-deleted mice showed a more robust increase in the proportion of type I fibers (Fig. 8A, 8B). Gastrocnemius muscle showed similar changes (data not shown). Notably, trained *Myog*-deleted mice showed a 91% increase in the proportion of type I fibers compared with untrained Myog-deleted mice; trained wild-type mice showed a 22% increase in the proportion of type I fibers compared with untrained wild-type mice, indicating that enhanced myofiber type remodeling occurred in trained *Myog*-deleted mice (Fig. 8B).

Exercise training produced a large increase in SDH activity in the gastrocnemius muscle of wild-type mice (Fig. 8C, 8D). However, trained *Myog*-deleted mice showed an even greater increase (Fig. 8C, 8D). Comparison of trained *Myog*-deleted and trained wild-type mice indicated that SDH activity in *Myog*-deleted mice was 66% greater (Fig. 8D). Myoglobin expression and SDH activity co-localized in trained mice, confirming that slow twitch fibers had increased in proportion to fast twitch fibers (data not shown). The increase in the proportion of type I fibers and in SDH activity indicated that compared to wild-type mice, long-term endurance trained *Myog*-deleted mice have enhanced oxidative metabolism and are inherently more prone to histological changes and fiber type remodeling.

### *Myog*-deleted mice have altered expression of genes involved in metabolism and signal transduction

Previous studies in our laboratory used differentiating wild-type and *Myog*-deleted adult satellite cells to identify genes whose expression was upregulated or downregulated in the absence of myogenin. We found that although *Myog*-deleted cultured satellite cells were able to fully differentiate into myotubes as efficiently as wild-type satellite cells, the absence of myogenin resulted in a substantial alteration in the expression of genes encoding proteins involved in cell signaling and cell fusion [9]. Notably, myogenin-dependent genes identified in differentiating satellite cells were a distinctly different set from those we had previously found in embryonic tongue muscle. To extend our gene expression profiling analysis to adult muscle, we used cRNA targets generated from the gastrocnemius muscle RNA of wild-type and *Myog*-deleted mice for hybridization to Affymetrix microarrays. Gastrocnemius muscle RNA was extracted within a day after the mice had been run to exhaustion in the high-intensity running regimen. The analysis identified differential expression of 30 downregulated genes and 11 upregulated genes whose expression was altered at least 1.4 fold. The differentially expressed genes were enriched for genes encoding proteins involved in signal transduction and metabolism (Table S1). Twelve genes were selected for validation using RT-qPCR (Fig. S9). These genes included Rho-related BTB domain-containing 1, a rho GTPase, a rho GTPase binding protein, a serine peptidase, glutathione peroxidase 3, pyruvate carboxylase, Nfatc2, zinc finger and SCAN domain-containing 21, Bmp5, macrophage activation 2-like, 3-hydroxybutyrate dehydrogenase, phosphofructokinase/fructose-bisphosphatase 3, and dihydropyrimidine dehydrogenase. The gene expression profiling analysis indicated that although skeletal muscle in adult *Myog-*deleted mice appeared morphologically identical to wild-type muscle, the muscle gene expression program was significantly modified. Many of the alterations in gene expression caused by the absence of myogenin were likely to be associated with the enhanced exercise capacity of *Myog*-deleted mice.

## Discussion

The results presented here identify new functions for myogenin in response to exercise stress and demonstrate that *Myog*-deleted adult mice possess an enhanced capacity for exercise soon after *Myog* deletion. In the absence of *Myog*, these mice exhibit increased aerobic capacity compared to their wild-type counterparts and utilize primarily oxidative metabolism for longer periods of time during exercise. *Myog*-deleted mice display increased levels of lactate build-up at exhaustion and are capable of further tapping into glycogen stores that wild-type mice do not access before reaching exhaustion. The data suggest that, in adult mice, the loss of myogenin abruptly alters metabolic processes, and thereby confers an enhanced capacity for exercise. When given access to a means of training over time, these shifts in metabolic processes translate into the acceleration of structural changes in the muscle fibers to induce enhanced shifts in fiber type. The results provide a direct demonstration that myogenin regulates metabolic homeostasis during adult life and may potentially negatively regulate exercise performance.

### *Myog* modulates exercise endurance by altering skeletal muscle metabolism

To avoid the effects of *Myog* deletion on postnatal body growth, we deleted *Myog* after mice had reached their adult size. In contrast to deleting *Myog* on the day after birth, we found that deletion of *Myog* from adult mice had few observable effects. Mice lacking *Myog* fed normally, exhibited increased cage activity by 1 year of age, and retained normal survival over a 2-year period of observation. While we detected an increase in cage activity in *Myog*-deleted, it is currently unclear whether this increase in activity produces the training benefits produced by more intense activities that use large muscle groups, increase energy expenditure, and stimulate cardiovascular function. *Myog*-deleted animals did not exhibit a difference in body weight within three months post-deletion, but weighed 9.4% less after one year. Silencing *Myog* expression during adult life in mice without noticeable consequences allowed us to pursue its potential role in adult skeletal muscle metabolism.

Surprisingly, within 2 weeks after *Myog* deletion, untrained mice performed significantly better in involuntary exercise running regimens than wild-type control mice. Under a low-intensity running regimen, *Myog*-deleted mice outperformed their wild-type counterparts. Enhanced aerobic endurance would typically be accompanied by measurable changes in fiber type composition, oxidative potential, metabolic enzyme activities, and altered expression of genes encoding enzymes involved in metabolism. Although we failed to find muscle enzymatic activity or histological evidence to support the observed enhanced aerobic endurance at the cellular level, we were able to detect changes in the expression of several genes that play a role in metabolism. The expression of Cox8b, a component of the mitochondrial electron transport chain, was increased by 84% in the absence of myogenin. The up regulation of this mitochondrial gene confirms the observed trend towards increased mtDNA and is likely associated with the enhanced oxidative capacity of *Myog*-deleted mice. UCP3 expression was decreased by 46%, further supporting an enhanced oxidative capacity. UCP3, which is primarily expressed in skeletal muscle mitochondria, has been suggested to be involved in mediating energy expenditure and is down regulated when oxidative capacity is high [18]. Increased expression of Ppar-g has been correlated with exercise training and increased physical activity [19]. We detected a 52% increase in Ppar-g expression, and its upregulation may be partially responsible for the increases in activity levels, oxidative capacity and exercise capacity of *Myog*-deleted mice. *Myog*-deleted mice exhibited better aerobic endurance and increased oxygen consumption associated with alterations in various metabolic genes, but without exhibiting the expected traditional changes on the cellular level.

*Myog*-deleted mice also performed better during high-intensity exercise. Subjecting the mice to uphill running at progressively faster speeds for 12 consecutive days demonstrated that *Myog*-deleted mice consistently performed better than wild-type mice and that the absence of myogenin did not cause a progressive decrease in performance from day to day. These differences in exercise endurance cannot be attributed to differences in body mass as *Myog*-deleted mice did not exhibit significant differences in weight until one year post-deletion. The results indicate that muscle maintenance and regeneration occur normally in exercising *Myog*-deleted mice and suggest that myogenin is not required for the maintenance of adult skeletal muscle.

Blood glucose measurements provided insight as to why *Myog*-deleted mice exhibit increased exercise endurance. Typical adaptations to endurance exercise involve a more efficient utilization of fatty acids and improved conservation of muscle and liver glycogen stores. Once muscle and liver glycogen stores are depleted, exercise performance sharply declines due to the inability of fatty acid metabolism to efficiently keep up with the ATP demands of the body [20]. While the increased depletion of muscle and liver glycogen, and the enhancement of exercise endurance could be independent of each other, *Myog*-deleted mice were nonetheless capable of exercise as blood glucose levels dropped significantly below levels normally associated with exhaustion. The ability to further deplete muscle and liver glycogen stores while continuing to exercise suggests that *Myog*-deleted mice have a higher exhaustion threshold. The enhanced reduction of blood glucose during low- and high-intensity exercise suggests that the lack of myogenin allows *Myog*-deleted mice to preferentially use liver glycogen stores as a source of energy for longer periods and indicates enhanced oxidative capacity.

Blood lactate measurements gave further insight into the basis of increased exercise endurance and fatigue resistance of *Myog*-deleted mice. Blood lactate levels traditionally build as exercise intensity increases. The concentration of lactate in the blood is directly associated with exercise fatigue and has been long argued to be a contributing factor [21], [22], [23], [24], [25], [26], [27]. During high-intensity exercise, blood lactate levels in *Myog*-deleted mice were not significantly increased beyond their baseline values at the wild-type exhaustion point. Lactate flux may be higher in *Myog*-deleted mice, allowing for efficient metabolism of lactate during earlier stages of exercise; during the later stages of exercise, a sudden increase in lactate concentration occurs as the lactate clearance rate reaches maximal capacity. The excessive production of blood lactate during the later stages of exercise indicated that *Myog*-deleted mice rely heavily on the glycolytic pathway for energy demand and that blood lactate does not limit their exercise endurance. Indeed, as others have shown, lactate production and efflux from the muscle may actually retard acidosis [16], [28] and contribute to the enhanced exercise performance of *Myog*-deleted mice. Indirect calorimetry showed that the lactate threshold, as determined by a sudden increase in VCO_2_ (RER>1), occurred immediately before *Myog*-deleted mice reached exhaustion, further suggesting that they were able to buffer H^+^, or generate less H^+^, prior to exhaustion. *Myog*-deleted mice might ultimately reach exhaustion under high-intensity exercise despite the acidosis associated with extreme lactate production. Acidosis is likely to limit exercise performance during high-intensity exercise before blood glucose is excessively depleted as it is during low-intensity exercise [20], [29]. Indeed, blood lactate levels were unchanged following low-intensity exercise in *Myog*-deleted mice, suggesting more efficient metabolism of lactate.

Taken together, these results suggest that the extreme exercise performance of *Myog*-deleted mice is ultimately governed by the availability of liver and muscle glycogen stores and by the possible performance-enhancing effects of lactate. A decreased sensitivity to the depletion of glycogen reserves, the enhanced capacity to clear lactate, and the ability to withstand extreme muscle acidosis may be the physiological basis for the enhanced exercise endurance of *Myog*-deleted mice.

Significantly decreased liver glycogen stores in exhausted *Myog*-deleted mice were expected and was consistent with the dramatic drop in blood glucose seen in *Myog*-deleted mice. The fact that *Myog*-deleted mice were able to significantly deplete their muscle glycogen stores while wild-type mice were not might provide further insight as to how the observed enhanced exercise capacity is achieved. The conversion of glycogen to lactate produces half as many pH-lowering protons in the muscle as the conversion of glucose to lactate [16]. The data suggest that *Myog*-deleted mice are not only able to significantly deplete another fuel source that wild-type mice cannot, but they are tapping into reserves that delay the onset of acidosis.

Indirect calorimetry indicated that the loss of *Myog* confers multiple qualities similar to those found in well-conditioned aerobic athletes. Elevated oxygen consumption likely enabled *Myog*-deleted mice to maintain the use of oxidative metabolism for significantly longer periods of time under the high-intensity exercise regimen, thus delaying acidosis. This further explains the basis for the observation that *Myog*-deleted blood lactate levels were much lower than wild-type at the time when wild-type mice reached exhaustion during high-intensity exercise. The observed trend towards increased mitochondria activity suggests that increased oxygen consumption of *Myog*-deleted mice is the result of a higher number of mitochondria.

In addition to *Myog*-deleted mice, mice harboring transgenes encoding proteins associated with energy metabolism and myofiber remodeling have been shown to possess increased exercise capacity. A transgenic line of mice that overexpresses the gene encoding phosphoenolpyruvate carboxykinase (PEPCK) in skeletal muscle was shown to increase cage activity and treadmill exercise capacity by re-patterning energy metabolism [30]. *PEPCK* transgenic mice also exhibited higher oxygen consumption, increased food intake, decreased body weight, and increased mitochondria in their muscles. Overall, *PEPCK* overexpression increased the citric acid cycle flux to generate more energy for muscle contraction during strenuous exercise. In contrast, transgenic mice overexpressing *PPAR-delta* in skeletal muscle demonstrated enhanced exercise performance via an increase in slow twitch fiber composition [31]. PPAR-delta is a transcription factor involved in regulating fat burning in adipose tissue and myofiber specification. The transcriptional co-activator PGC-1 alpha also drives slow twitch fiber formation [32]. *PGC1-alpha* is expressed in brown fat and skeletal muscle and activates mitochondrial biogenesis. When *PGC1-alpha* is expressed in fast twitch fibers, genes involved in oxidative metabolism are activated [32]. Lastly, several studies from the Olson laboratory have demonstrated that myofiber remodeling is mediated by HDAC4 and Mef2 transcription factors that respond to calcineurin signaling. Acting through calcineurin signaling, calsarcin-2, a muscle-specific protein of the sarcomeric Z-disk, modulates exercise performance and alters skeletal muscle fiber type composition [33]. In all of these mouse genetic models, enhanced exercise performance is associated with alterations in mitochondrial activity, muscle fiber specification, and calcium signaling. In our study, the enhanced exercise endurance of *Myog*-deleted mice may involve alternative pathways to the ones described above.

Enhanced exercise endurance in untrained mice lacking myogenin was not associated with a change in myosin ATPase fiber type, myosin isoform expression, myofiber oxidative potential, the metabolism of fatty acids, or increased food consumption. The ability to shift the metabolic profile of skeletal muscle fibers without a change in myosin heavy chain expression is well-documented [13]. Because fiber type remodeling is a long-term adaptation [34], changes in the expression levels of metabolic proteins in *Myog*-deleted mice may be responsible for the enhanced exercise capacity observed within 2 weeks of *Myog* deletion. Notably, the overexpression of myogenin shifts the muscle enzymatic activity from glycolytic towards oxidative metabolism, independent of a change in fiber type [13]. Although myogenin was shown to be an oxidation-promoting factor, these studies did not demonstrate the physiological effects of myogenin overexpression, specifically with regards to enhanced exercise capacity and increased oxygen consumption. While the presence of myogenin leads to increased oxidative enzyme activity and mediates the effect of innervation on slow twitch muscle fibers, the absence of myogenin does not necessarily preclude the possibility of increased oxidative capacity, as shown by our results. The data support the hypothesis that enhanced exercise capacity is the result of increased oxidative and glyocolytic metabolism.

The ability of mice lacking myogenin to outperform wild-type mice during running exercise raises the seemingly paradoxical question: Why does myogenin expression persist in adult skeletal muscle? Myogenin is not required for viability, and deletion of *Myog* is in fact advantageous to exercise endurance. One possible explanation for *Myog* expression during adult life is that myogenin is more highly expressed in populations of mice that are dependent on environmental adaptations. Over many years and generations away from natural predators, common laboratory strains of mice may experience less selective pressure towards the adulthood silencing of myogenin, thus leading to the continued expression of *Myog* and increased development of slow-oxidative fibers after training. Myogenin may exhibit lower levels of expression in some wild populations of mice whose environment requires rapid responses to avoid predators and acquire food. A second but not mutually exclusive possibility is that myogenin is essential for survival. *Myog* deletion results in an excessive depletion of blood glucose upon intense exercise. The body's natural ability to sense hypoglycemia and react by ceasing exercise activity would be beneficial in most environments, given that long-term effects of hypoglycemia increase overall morbidity and mortality [35], [36]. Environmental pressures would drive such natural selection. The presence of myogenin during adult life could contribute to feedback mechanisms ultimately allowing the body to cease excessive skeletal muscle activity in favor of blood glucose conservation. Furthermore, recent studies indicate myogenin is an essential activator of muscle atrophy [10]. Consistent muscle mass is achieved by a balance between the rates of protein synthesis and degradation. Through the activation of E3 ubiquitin ligases, myogenin may play an instrumental role in maintaining this balance by promoting proteolysis.

### Enhanced adaptation to exercise training in *Myog*-deleted mice

Fast- to slow-twitch fiber-type transition and increased oxidative capacity are well-documented, long-term adaptive responses to exercise training [37], [38], [39], [40], [41], [42], [43]. By providing unrestricted access to voluntary exercise, we were able to determine that *Myog*-deleted mice were significantly better adapted to exercise training than wild-type mice. As expected, wild-type mice exhibited enhanced exercise endurance when they were subjected to involuntary high-intensity exercise following the 6-month voluntary training period. Unexpectedly, trained *Myog*-deleted mice exhibited a superior adaptive response to exercise training under similar conditions. The effects of exercise training coupled to the deletion of myogenin appeared to be synergistic, because sedentary *Myog*-deleted mice were comparable to trained wild-type mice in their exercise capacity.

The superior exercise endurance of trained *Myog*-deleted mice was associated with a comparatively higher proportion of both slow-twitch fibers and SDH-positive fibers than in trained wild-type control mice. The further increased proportion of the slow-twitch fibers and oxidative capacity may allow *Myog*-deleted mice to efficiently conserve muscle and liver glycogen stores by more efficiently utilizing fatty acids during low-intensity exercise. As exercise intensity progresses, a more efficient glycolytic metabolism and excessive depletion of glycogen reserves support the energy demands of high-intensity exercise.

### Myogenin's role as a transcription factor in energy metabolism

In wild-type mice, we observed a significant correlation between the duration of low-intensity exercise and the abundance of *Myog* muscle transcripts immediately following exhaustion. Furthermore, we also detected a strong trend towards increased *Myog* expression in mice immediately after low-intensity exercise. Mice that ran the least displayed the lowest levels of *Myog* whereas mice that ran the most exhibited the highest levels of *Myog*. These data suggest that *Myog* is minimally expressed in normal adult muscle, but may be upregulated after long periods of exercise proportional to the amount of running performed. The normal function of *Myog* during exercise is likely to help coordinate the upregulation of satellite cell-expressed genes following significant muscle catabolism; these genes would then help facilitate muscle repair. As the deletion of *Myog* does not compromise their ability to outperform wild-type mice on a daily basis, we suggest that *Myog* is not essential for normal muscle function or repair following long periods of exercise.

**Figure 9:**
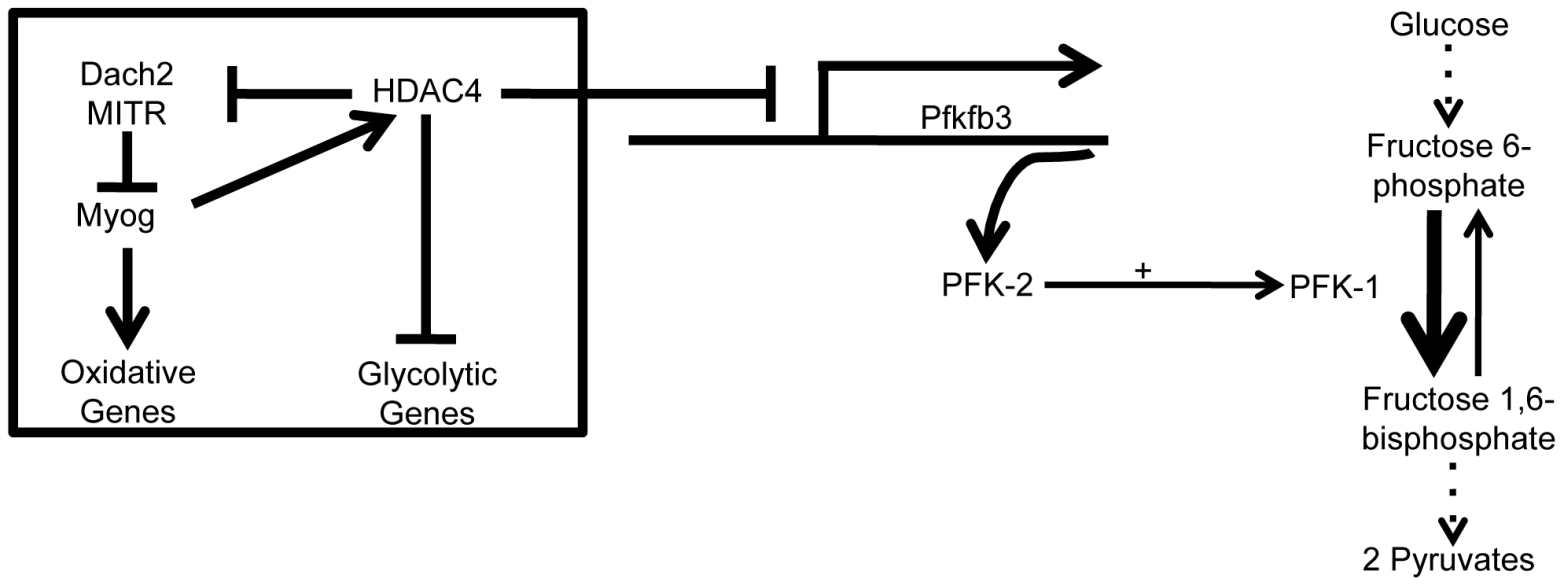
Proposed model for myogenin's role as a transcription factor regulating the expression of genes associated with skeletal muscle energy metabolism. Myogenin positively regulates the expression of genes involved in oxidative metabolism and represses genes involved in glycolytic metabolism through HDAC4 activity. *Pfkfb3*, a gene involved in a rate-limiting step of glycolysis, converts F6P to F2,6BP, which positively regulates the activity of PFK-1. PFK-1 is a dual-activity enzyme that interconverts F6P and F1,6BP, directly regulating glycolytic flux. Pfkfb3 expression is upregulated in the absence of myogenin, thereby increasing PFK-1 activity and enhancing glycolytic flux.

Microarray analysis identified a number of genes whose expression was altered in skeletal muscle of exercise-trained *Myog*-deleted mice. Of particular note were those encoding proteins associated with signal transduction and metabolism. For example, *Pcx* (pyruvate carboxylase), whose expression was 20% lower in *Myog-*deleted mice, is involved in the first step of gluconeogenesis and plays a major role in both carbohydrate and lipid metabolism [44]. Pcx catalyzes an anaplerotic reaction, replenishing oxaloacetate within the citric acid cycle to maintain efficient oxidative metabolism [44]. A reduction in Pcx activity might result in enhanced glycolytic metabolism by partially shunting pyruvate away from the citric acid cycle, thus contributing to the increase in blood lactate levels. However, because Pcx deficiency is usually detrimental [45], [46], [47], the significance of its downregulation in *Myog*-deleted mice is unclear. *Pfkfb3* (phosphofructokinase/fructose-bisphophatase) encodes PFK-2, a protein involved in a rate-limiting, reversible step of the glycolytic pathway [48]. PFK-2 regulates the activity of PFK-1 and ultimately regulates glycolytic flux. Although PFK-2 is a dual activity enzyme that promotes both glycolysis and gluconeogenesis, this particular isoform has a 400∶1 tendency to enhance glycolytic flux in skeletal muscle [48]. *Pfkfb3* expression increases by 80% in *Myog*-deleted mice, and this increase may be involved in the mechanisms conferring enhanced exercise capacity. Indeed, myogenin has been previously shown to indirectly inhibit genes involved in the glycolytic pathway. Myogenin positively regulates genes involved in oxidative metabolism and represses genes involved in glycolytic metabolism through HDAC4 activity [49]. The upregulation of *Pfkfb3* suggests a model in which glycolytic metabolism may be partially enhanced by *Myog* deletion, mediated through a release of *Pfkfb3* inhibition (Fig. 9).

## Materials and Methods

### Ethics Statement

All experimental procedures described in this study followed the U.S. Public Health Service Policy of Humane Care and Use of Laboratory Animals and were approved by the Institutional Animal Care and Use Committee at The University of Texas MD Anderson Cancer Center and Baylor College of Medicine (ACUF ID#: 01-92-00237).

### *Myog*-deleted mice

For this study, we used mice that were homozygous for the *Myog^flox^* allele and hemizygous for the CAGGCre-ER transgene [50]. *Myog*-deleted mice were from a mixed background of C57/BL/6 and 129 strains. Genomic deletion of the floxed *Myog* allele was achieved in 8 to 12 week-old mice (by which time the body mass growth had significantly tapered off) by a single intraperitoneal injection of tamoxifen in corn oil at a concentration of 10 mg/40 g body weight. Genomic DNA deletion was determined 2 weeks following the deletion of Myog via qPCR. Deletion was initially tested in tail clippings and later confirmed in muscle tissue. To measure deletion efficiency, we used ABI Power SYBR and the following primers: Myog-LoxP-Fwd (5′–CCG GGT AGG AGT AAT TGA AAG GA–3′) and Myog-LoxP-Rev (5′–GCC GTC GGC TGT AAT TTG AT–3′). An ABI 7500 Fast Real Time PCR System was used to perform qPCR analysis using default conditions (95°C×10 min, 40 cycles of 95°C×15 s, 60°C×60 s). Unless otherwise noted, all experiments were performed on mice between 13 and 15 months of age, approximately one year after the deletion of *Myog*. The reduction of *Myog* transcripts was determined using Taqman RT-qPCR in muscle tissue collected immediately after euthanasia.

### Determination of exercise capacity

Involuntary treadmill running regimens used protocols from previously published reports [30]. Adult wild-type and *Myog*-deleted mice were run on a Exer-3/6 rodent treadmill (Columbus Instruments). The treadmill is equipped with a rear electrical stimulus grid set to deliver 0.2 mA. Mice were determined to have reached the point of exhaustion when they made contact with the grid for a period of 10 seconds. The stimulus was then turned off in the lane in which the mouse had run to exhaustion. The low-intensity exercise regimen consisted of mice warming up for 30 minutes at 10 m/minute at 0° incline. The speed was then increased to 20 m/minute at 0° incline, and the mice were run until exhaustion. The high-intensity exercise regimen consisted of mice warming up for 10 minutes at 20 m/minute at a 0° incline. The speed was then increased by 2 m/minute every 2 minutes while remaining at a 10° incline until exhaustion. Exercise duration, distance, and maximum speed were recorded at exhaustion.

For the voluntary exercise regimen, cages containing exercise wheels were built in-house. Exercise wheels were suspended in the center of the cage and held in place by wires fastened to the cage top and base. A single mouse was housed in each cage. Running wheels were connected to sensors that recorded time, average and maximum speed, and distance run.

### Determination of mtDNA:nDNA Ratio

Genomic DNA was prepared as described previously in [51]. Briefly, muscle and liver was homogenized using a Dounce homogenizer in lysis buffer [10 mM Tris-HCl (pH 8.0), 1 mM EDTA, and 0.1% SDS]. Proteinase K was added and lysates were incubated at 55°C for 3 hours. Lysates were vortexed and pelleted by centrifugation (8000 g for 15 min) and the supernatant was collected. Phenol-chloroform DNA extraction was then performed. qPCR analysis was used to quantify the proportion of mtDNA:nDNA in each sample. The cytochrome c oxidase subunit I (CO1) gene of the mtDNA and NDUFV1 nDNA gene were amplified with the following primers: CO1-Fwd (5′TGC TAG CCG CAG GCA TTA C3′), CO1-Rev (5′–GGG TGC CCA AAG AAT CAG AAC–3′), NDUFV1-Fwd (5′–CTT CCC CAC TGG CCT CAA G–3′), NDUFV1-Rev (5′–CCA AAA CCC AGT GAT CCA GC–3′). ABI Power SYBR Master Mix was used to perform mtDNA and nDNA quantification. An ABI 7500 Fast Real Time PCR System was used to perform qPCR analysis using default conditions (95°C×10 min, 40 cycles of 95°C×15 s, 60°C×60 s).

### Determination of blood lactate and blood glucose concentrations

Blood lactate concentrations were determined on tail vain blood samples using a Lactate Pro blood lactate test meter (Arkray USA) in resting mice and within 30 seconds of exhaustion. Blood glucose levels were determined on tail vain blood samples using a Precision Xtra glucometer in resting mice and within 30 seconds of exhaustion (Abbott Labs). Experiments where blood glucose and lactate were determined in *Myog*-deleted mice at the stage that wild-type mice had attained exhaustion were performed using the same group of *Myog*-deleted mice that had previously run to exhaustion. Wild-type exhaustion time was established from the average of three measurements performed on separate days in wild type mice.

### Biochemical determination of liver and muscle glycogen concentration

Soleus and EDL muscle, and liver were immediately harvested and flash frozen from euthanized wild-type and *Myog*-deleted mice. Mice used for glycogen determination were non-exercised for a minimum of one week and euthanized within 4 hours after the beginning of the light cycle. An abbreviated protocol followed from MBL International Corporation (www.mblintl.com) included the following steps: tissues were isolated and snap frozen in liquid nitrogen. Samples were homogenized in dH_2_O, boiled for 5 minutes, and centrifuged at 13,000 rpm for 5 min. A glucoamylase hydrolysis enzyme mix was then added to the supernatants. A master mix of development buffer, development enzyme mix, and OxiRed probe was then added to the samples and left to incubate at room temperature protected from light for 30 minutes. Samples were then measured colorimetrically using a spectrophotometer at 570 nm. Non-hydrolysis control readings were then subtracted from the experimental raw values and glycogen concentration was determined from a standard curve.

### Indirect Calorimetry, food Intake, spontaneous activity and dody composition determination

One-year old wild-type and *Myog*-deleted mice were individually caged in a Comprehensive Laboratory Animal Monitoring System (Columbus Instruments) for a total of three days during which time food intake, O_2_ consumption and CO_2_ production (from which energy expenditure and RER was calculated), and activity were continuously monitored. The first day's data obtained to allow the mice to adjusted to their new environment, Data was evaluated on the last two days only. On the fourth day the monitoring was continued for a further eight hours from the time the lights were on but with access to food denied. The average of the two minimal values for VO_2_ that were associated with no cage activity during the last four hours was defined as the resting metabolic rate.

On completion of the metabolic measurements, the mice were weighed and their fat and lean masses determined by quantitative nuclear magnetic resonance using an EchoMRI-100™ instrument (Echo Medical Systems).

To determine O_2_ consumption and CO_2_ production during exercise, wild-type and *Myog*-deleted mice taken from those used for the metabolic measurements were analyzed. Mice were run individually on a modular treadmill (Columbus Instruments). After familiarizing each mouse to the treadmill, on a subsequent day, mice were subjected to the high-intensity exercise regimen until they reached exhaustion. O_2_ consumption, CO_2_ production, RER, and energy expenditure were recorded every 15 seconds.

### Histology

Gastronemius, soleus, and EDL muscles were dissected and snap frozen in liquid nitrogen-cooled isopentane. Fresh-frozen sections (10 to 12 µm) were then prepared on a Cryostat. To perform fiber typing, we used a protocol available from Washington University Neuromuscular Disease Center. Briefly, histological sections were incubated for 5 minutes in pre-incubation solution, pH 4.6 (5.0 ml barbital acetate solution [1.47 g sodium barbital, 0.97 g sodium acetate, 50 ml final volume in H_2_O], 10 ml 0.1N HCl, 4.0 ml H_2_O), rinsed with water and then incubated for 25 minutes in ATP solution, pH 9.4 (60 mg ATP powder, 6.0 ml 0.1 M sodium barbital, 21.0 ml H_2_O, 3.0 ml 0.18 M calcium chloride). The sections were then washed with three exchanges of 1% CaCl_2_ for 10 minutes, 2% CoCl_2_ for 10 minutes, four exchanges of a 1∶20 solution of 0.1 M sodium barbital, and five exchanges with water. The sections were then exposed to 2% ammonium sulfide for 20–30 seconds, washed with five exchanges of water, dehydrated in ascending alcohols, cleared with xylene, and mounted with Canada balsam.

To determine SDH activity, we used a modified protocol from the University of Nottingham Medical School [52]. Sections were incubated for 5 minutes in a medium containing 0.2 ml of 500 mM sodium succinate, 0.7 mg of phenazine methosulfate, and 2.0 ml of NBT solution (6.5 mg KCN, 185 mg EDTA, 100 mg nitroblue tetrazolium, 100 ml of 100 mM phosphate buffer pH 7.6 [12 ml of solution A [1.36 g KH_2_PO_4_ in 100 ml of H_2_O] and 88 ml of solution B [1.42 g Na_2_HPO_4_/100 ml H_2_O]]). The sections were then washed, fixed for 15 minutes in formalin, dehydrated in ascending alcohols, cleared with xylene, and mounted. Values for each mouse were averaged from the analyses of two 1 mm^2^ fields

### Affymetrix gene microarray hybridization and analysis

RNA was isolated from hindlimb muscle of trained wild-type and *Myog*-deleted mice. One hundred nanograms of total RNA from each sample were used for preparing hybridization probes. Three independent samples were used in standard Affymetrix protocols to yield biotin labeled cRNA fragments, which were hybridized to Mouse Genome 430 2.0 GeneChips (Affymetrix). The hybridized arrays were scanned using Affymetrix GCOS software with Robust Multi-chip Averaging normalization [53]. Unpaired two-class *t*-tests were performed and significance cutoffs were set at *P<*0.05. From the filtered data set, all genes changing >1.4 fold were kept for further analysis. The data discussed in this publication are MIAME compliant have been deposited in NCBI's Gene Expression Omnibus [54] and are accessible through GEO Series accession number GSE22046, (http://www.ncbi.nlm.nih.gov/geo/query/acc.cgi?acc=GSE22046).

### RNA isolation and reverse transcriptase-quantitative PCR

RNA was isolated from dounce-homogenized mouse skeletal muscle or liver using the Invitrogen Trizol kit. Reverse transcription reactions were performed used Applied Biosystems RNA to cDNA kits. Power SYBR green, TaqMan master mix, and Taqman gene expression assays were purchased from Applied Biosystems (Foster City, CA) and used to determine cDNA abundance for each gene analyzed. An ABI 7500 Fast Real Time PCR System was used to perform qPCR analysis using default conditions (95°C×10 min, 40 cycles of 95°C×15 s, 60°C×60 s). Gene expression was normalized to the Gapdh reference gene and analysis was performed via the ddCT method. Taqman hydrolysis primer and probe gene expression assays were ordered with the following assay IDs: Myog, Assay ID: Mm00446194_m1; Gapdh, Assay ID: Mm03302249_g; UCP2, Assay ID Mm00627599_m1; UCP3, Assay ID Mm00494077_m1; Cox8b, Assay ID Mm00432648_m1; Glut4, Assay ID Mm00436615_m1; CD36, Assay ID Mm01135198_m1; Acc2, Assay ID Mm01204691_m1; ppar-a; Assay ID Mm00627559_m1; ppar-g, Assay ID Mm00440945_m1; Cpt1, Assay ID Mm00550438_m1; Rfx7, Assay ID Mm01146805_m1; Nfatc2, Assay ID Mm00477776_m1; RhoBTB1, Assay ID Mm01143659_m1; Rhpn2, Assay ID Mm00518451_m1; Htra1, Assay ID Mm00479887_m1; Gpx3, Assay ID Mm00492427_m1; Zscan21; Assay ID Mm00442147_m1; Pcx, Assay ID Mm00500992_m1; Bmp5, Assay ID Mm00432091_m1; Pfkfb3, Assay ID Mm00504650_m1; Mpa2l, Assay ID Mm00843395_m1; Dpyd, Assay ID Mm00468111_m1; Bdh1, Assay ID Mm00558330_m1. Further details may be obtained by contacting the corresponding author.

### Determination of enzyme activities

To determine the activities of metabolic enzymes, we used soleus, EDL, and plantaris muscles from wild-type and *Myog*-deleted mice. Briefly, fast and slow twitch muscles (EDL and soleus, respectively) were isolated and snap frozen in liquid nitrogen. Muscles were homogenized in stabilizing medium (50% glycerol, 20 mM phosphate buffer, pH 7.4, 5 mM beta-mercaptoethanol, 0.5 M EDTA, 0.02% bovine serum albumin) [13] at a 1∶100 dilution based on wet weight. The enzyme activities measured included pyruvate kinase, myokinase, **L**-lactate dehydrogenase, glycogen phosphorylase, β-hydroxyacyl-CoA-dehydrogenase, glycerol-3-phosphate dehydrogenase and malate dehydrogenase.

Cuvettes were prepared with the buffer and substrates required for the particular assay and monitored with a spectrophotometer at A_340 nm_ until constant. Diluted homogenates were then added to each cuvette in duplicate and monitored with continuous spectrophotometric rate determination, recording the change in A_340 nm_ for approximately 10 minutes at 25°C. The change in A_340 nm_ per minute was determined using the maximum linear rate for both the test and the blank. Activities are presented as millimoles per kilogram per minute. Detailed protocols are available from the Sigma Aldrich Enzyme Explorer Assay Library.

### Morphometric analysis of muscle fibers

Corresponding dorsomedial fields from each wild-type and *Myog*-deleted mouse were selected from the gastrocnemius and soleus muscles for SDH and fibertyping analyses. Fibers were then counted in a blind study using NIH ImageJ Software. [55]

### Statistical analysis

Statistical analyses were performed using two-tailed pooled Student's *t*-tests in Microsoft Excel 2008. A confidence level of *P<*0.05 was considered to be statistically significant. Error bars represent one standard deviation from the mean. To analyze increased training response, a general linear model was fitted to evaluate the effects of marker (wild-type versus *Myog*-deleted groups), training status, and their interaction on dependent variable distance. Analysis indicated that, compared with the marker wild-type group, the difference in distance between the training and non-training groups was significantly different.

## Supporting Information

Figure S1 Efficient deletion and viability of adult Myog-deleted mice. (A) qPCR revealed efficient 96% deletion of Myog genomic DNA in adult mice following tamoxifen treatment (n = 10 wild-type control, n = 12 Myog-deleted) (A). (B) At one year of age, hindlimb muscle of Myog-deleted mice exhibited dramatically reduced expression of Myog, less than 1% of wild-type control mice, as shown by RT-qPCR (n = 3 per group). (C) One-year-old Myog-deleted mice weighed the same as wild-type control mice (n = 11 per group). Blue bars indicate wild-type control values; red bars indicate Myog-deleted values. Error bars represent one standard deviation. *p<0.05.
(0.14 MB TIF)

Figure S2 Low- and high-intensity involuntary running regimens. Low-intensity exercise running (blue line) consisted of a 30 minute warm up period at 10 M/min followed by running at 20 M/min until exhaustion. High-intensity running (red line) consists of a 10 minute warm up period at 10 m/min followed by running at an additional 2 m/min every 2 minutes until exhaustion. Exhaustion was defined as when mice preferred to contact the electrical stimulus grid for 10 seconds rather than run.
(0.10 MB TIF)

Figure S3 Myog-deleted mice exhibit enhanced exercise capacity shortly after the deletion of Myog. Young adult mice were subjected to high-intensity running ten days after tamoxifen treatment. (A) Myog-deleted mice exhibited a 1.4-fold increase in high intensity exercise endurance relative to wild-type control mice (Myog-deleted, 547 m, wild-type 402 m, n = 24 mice/group). (B) Myog-deleted males ran 1.6-fold farther than wild-type males (Myog-deleted 539 m, wild-type 336 m, n = 12 mice/group). (C) Myog-deleted females showed a strong trend (p = 0.06) towards running nearly 20% farther than wild-type females (Myog-deleted 555 m, wild-type 468 m, n = 12 mice/group). Mice in this experiment were two to four months of age, with an average of three months of age. Blue bars indicate wild-type control values; red bars indicate Myog-deleted values. Error bars represent one standard deviation. *p<1x10-4.
(0.13 MB TIF)

Figure S4 Myog expression increases in response to low-intensity exercise in wild type mice. (A) After running to exhaustion under the low-intensity regimen, wild-type mice exhibited a strong trend toward increased Myog transcript expression, but not after high-intensity running to exhaustion. (B) A directly proportionate relationship exists in wild-type mice between Myog transcript expression and distance run to exhaustion. Mice running greater distances possess increased Myog levels (R2 = 0.905 and Pearson correlation P = 0.013). Sedentary, n = 6; Low-intensity exhaustion, n = 6; High-intensity exhaustion, n = 6. Green bar indicates Myog expression values for sedentary group; orange bar indicates Myog expression values for sedentary group Myog-deleted values. Error bars represent one standard deviation.
(0.13 MB TIF)

Figure S5 Soleus and liver glycogen concentration. Soleus (A) and liver (B) glycogen concentrations were significantly depleted in Myog-deleted mice (63% and 51%, respectively), but decreases in wild-type soleus and liver (51% and 21%, respectively) were less dramatic and not statistically significant. Myog-deleted, n = 6; wild-type, n = 6. Blue bars indicate wild-type control values; red bars indicate Myog-deleted values. Error bars represent one standard deviation. *P<0.05.
(0.17 MB TIF)

Figure S6 Mitochondrial abundance in gastrocnemius muscle of wild-type and Myog-deleted mice. Myog-deleted mice exhibited a strong trend toward an increased ratio of mitochondrial DNA to genomic DNA over the wild-type group (Myog-deleted, n = 6; wild-type, n = 6). Blue bars indicate wild-type control values; red bars indicate Myog-deleted values. Error bars represent one standard deviation.
(0.06 MB TIF)

Figure S7 Myog-deleted mice exhibit normal expression of fatty-acid metabolism, mitochondrial function, and GLUT4 genes in gastrocnemius muscle. Taqman RT-qPCR of selected genes that play a role in metabolism include: Acc2 (acetyl-coenzyme A carboxylase beta, biosynthesis of fatty acids), CD36 (cluster of differentiation 36, fatty acid and glucose metabolism), Cox8b (cytochrome C oxidase subunit 8b, mitochondrial electron transport chain, oxidative ATP production), Cpt-1 (carnitine palmitoyltransferase I, transport of long chain fatty acids across mitochondrial membrane), Glut4 (glucose transporter type 4, glucose uptake), Ppar-a (peroxisome proliferator-activated receptor alpha, nuclear receptor, regulation of fatty acid storage and glucose metabolism), Ppar-g (peroxisome proliferator-activated receptor gamma, nuclear receptor, regulation of fatty acid storage and glucose metabolism), UCP2 (mitochondrial uncoupling protein 2, oxidative phosphorylation), and UCP3 (mitochondrial uncoupling protein 3, oxidative phosphorylation). Of the oxidative metabolism genes tested, the expression of Cox8b and Ppar-g was determined to be significantly increased in gastrocnemius muscle of Myog-deleted mice, (84% and 52%, respectively). UCP3 and Glut4 expression levels were significantly decreased (46% and 37%, respectively). Blue bars indicate wild-type control values; red bars indicate Myog-deleted values. (Myog-deleted, n = 6; wild-type, n = 6). Error bars represent one standard deviation. *p<0.05, **p<0.01
(0.14 MB TIF)

Figure S8 Myog-deleted mice exhibit normal oxidative and glycolytic metabolic enzyme activities in the soleus and extensor digitorum longus (EDL) muscles. (A) Glycolytic enzyme activities in the soleus muscle. (B) Glycolytic enzyme activities in the EDL muscle. (C) Oxidative enzyme activities in the soleus muscle. (D) Oxidative enzyme activities in the EDL muscle. Blue bars indicate wild-type control values; red bars indicate Myog-deleted values. (Myog-deleted, n = 4; wild-type, n = 4). Error bars represent one standard deviation.
(0.38 MB TIF)

Figure S9 Myogenin regulates genes involved in metabolism and signal-transduction during adult life. Taqman RT-qPCR validation was performed on selected differentially expressed genes in adult Myog-deleted gastrocnemius muscle. Genes: RhoBTB1 (rho-related BTB domain containing 1, rho GTPase), Rhpn2 (rhophilin, rho GTPase binding protein), Htra1 (serine peptidase), Gpx3 (glutathione peroxidase 3), Pcx (pyruvate carboxylase), Nfatc2 (nuclear factor of activated T-cells 2), Zscan21 (zinc finger and SCAN domain containing 21), Bmp5 (bone morphogenetic protein 5), Mpa2l (macrophage activation 2 like), Bdh (3-hydroxybutyrate dehydrogenase), Pfkfb3 (phosphofructokinase/fructose-bisphosphatase 3), and Dpyd (dihydropyrimidine dehydrogenase). Blue bars indicate wild-type control values; red bars indicate Myog-deleted values. Error bars represent one standard deviation. *p<0.05, **p<0.01.
(0.13 MB TIF)

Table S1 Gene expression profiling of Myog-deleted mice reveals differentially expressed genes involved in signal transduction and metabolism.
(0.09 MB DOC)
