# Supplementary figures and images for: Myogenin Regulates Exercise Capacity and Skeletal Muscle Metabolism in the Adult Mouse
Source: PLoS One. 2010 Oct 22;5(10):e13535. doi: 10.1371/journal.pone.0013535 (PMC2962629; doi:10.1371/journal.pone.0013535)

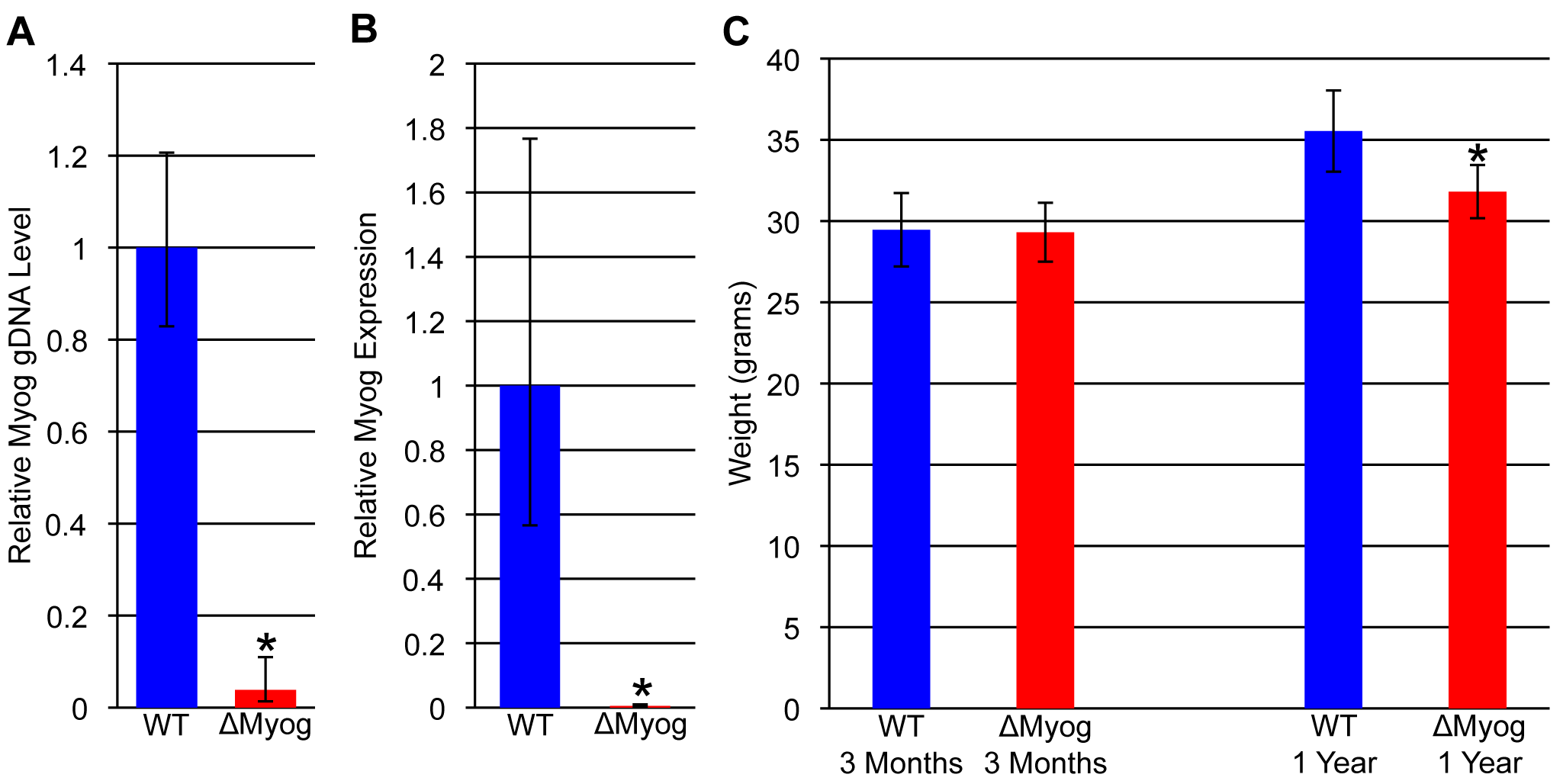

Supplement: Figure S1 — Efficient deletion and viability of adult Myog-deleted mice. (A) qPCR revealed efficient 96% deletion of Myog genomic DNA in adult mice following tamoxifen treatment (n = 10 wild-type control, n = 12 Myog-deleted) (A). (B) At one year of age, hindlimb muscle of Myog-deleted mice exhibited dramatically reduced expression of Myog, less than 1% of wild-type control mice, as shown by RT-qPCR (n = 3 per group). (C) One-year-old Myog-deleted mice weighed the same as wild-type control mice (n = 11 per group). Blue bars indicate wild-type control values; red bars indicate Myog-deleted values. Error bars represent one standard deviation. *p<0.05. (0.14 MB TIF) [file pone.0013535.s001.tif]

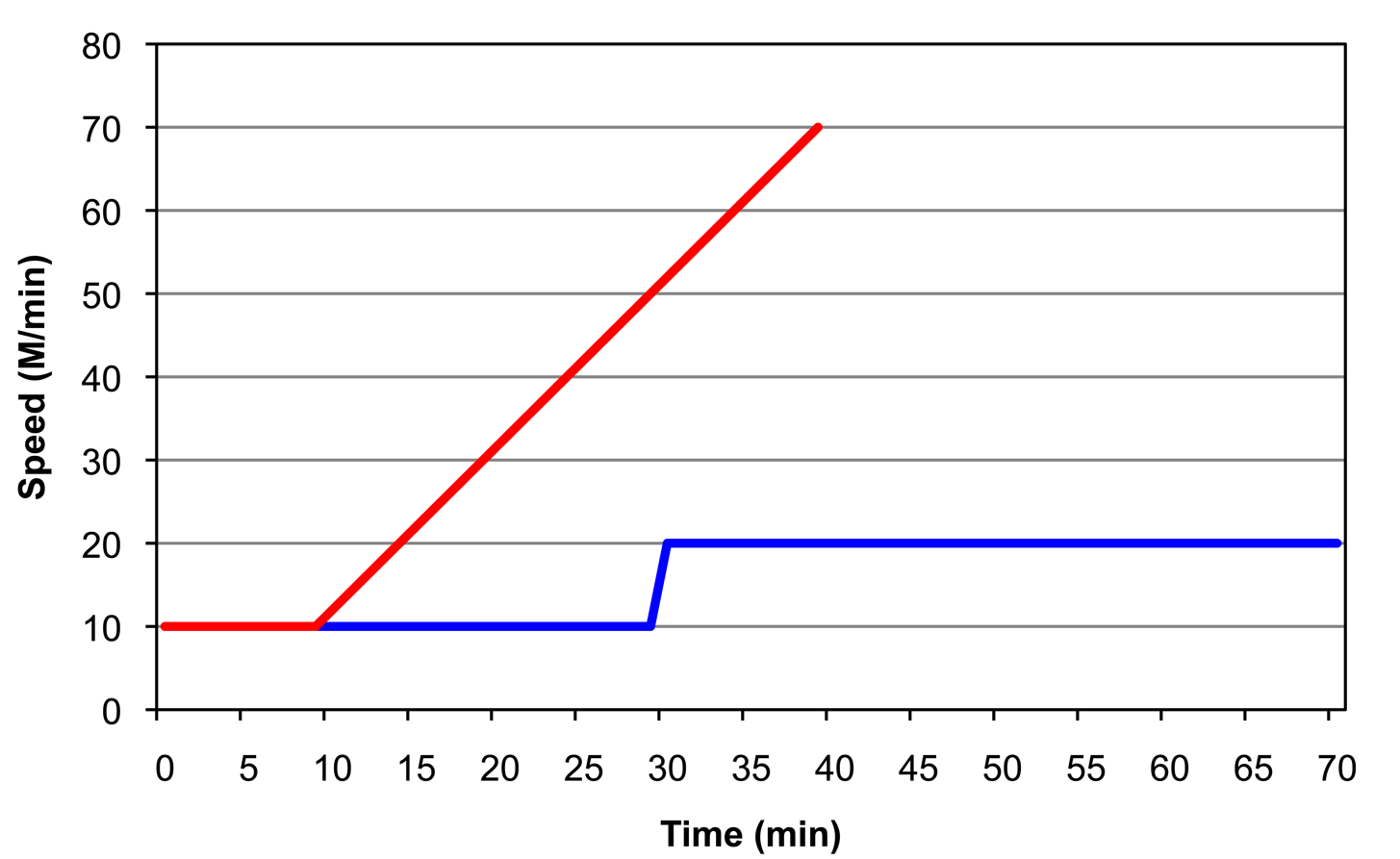

Supplement: Figure S2 — Low- and high-intensity involuntary running regimens. Low-intensity exercise running (blue line) consisted of a 30 minute warm up period at 10 M/min followed by running at 20 M/min until exhaustion. High-intensity running (red line) consists of a 10 minute warm up period at 10 m/min followed by running at an additional 2 m/min every 2 minutes until exhaustion. Exhaustion was defined as when mice preferred to contact the electrical stimulus grid for 10 seconds rather than run. (0.10 MB TIF) [file pone.0013535.s002.tif]

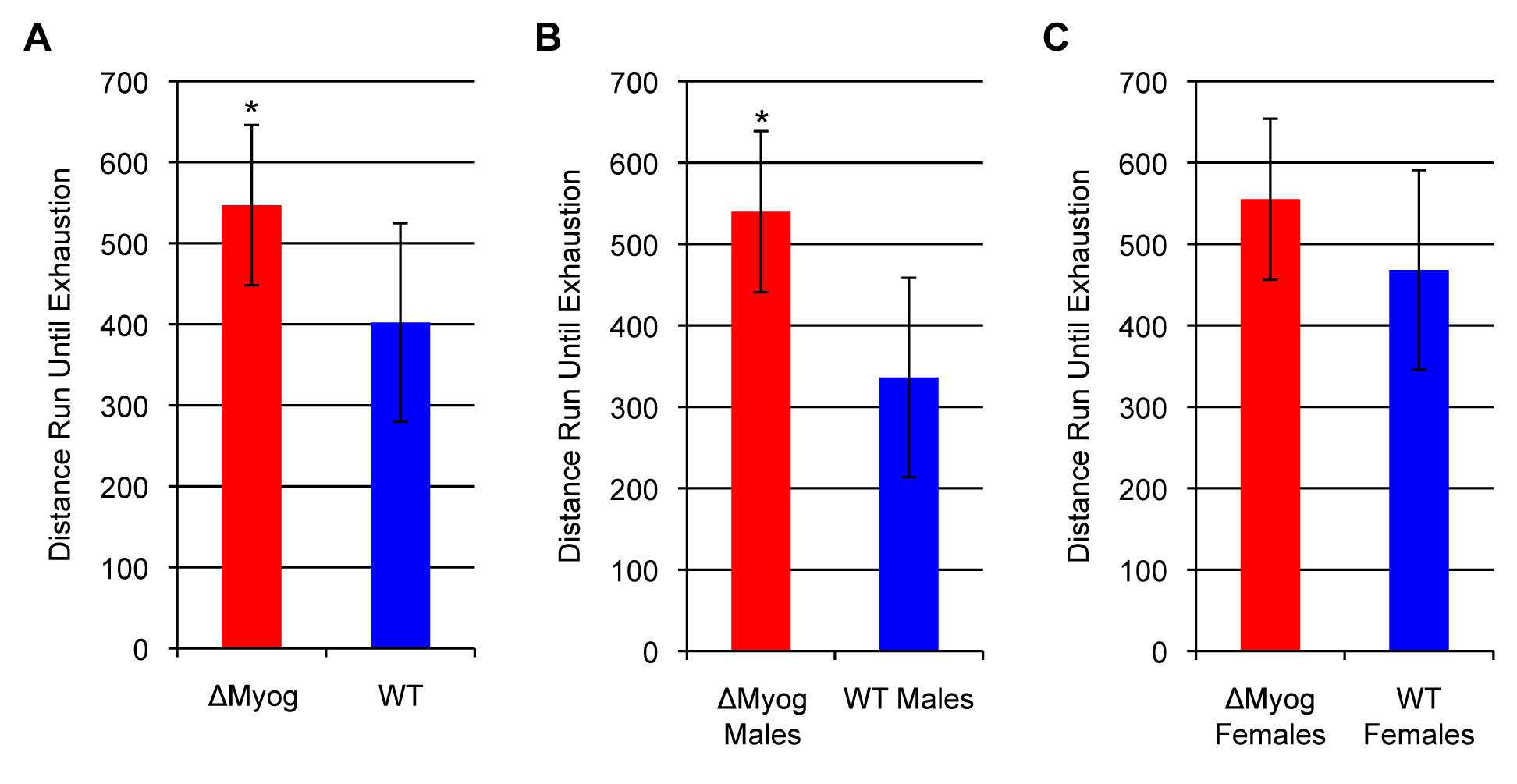

Supplement: Figure S3 — Myog-deleted mice exhibit enhanced exercise capacity shortly after the deletion of Myog. Young adult mice were subjected to high-intensity running ten days after tamoxifen treatment. (A) Myog-deleted mice exhibited a 1.4-fold increase in high intensity exercise endurance relative to wild-type control mice (Myog-deleted, 547 m, wild-type 402 m, n = 24 mice/group). (B) Myog-deleted males ran 1.6-fold farther than wild-type males (Myog-deleted 539 m, wild-type 336 m, n = 12 mice/group). (C) Myog-deleted females showed a strong trend (p = 0.06) towards running nearly 20% farther than wild-type females (Myog-deleted 555 m, wild-type 468 m, n = 12 mice/group). Mice in this experiment were two to four months of age, with an average of three months of age. Blue bars indicate wild-type control values; red bars indicate Myog-deleted values. Error bars represent one standard deviation. *p<1x10^-4^. (0.13 MB TIF) [file pone.0013535.s003.tif]

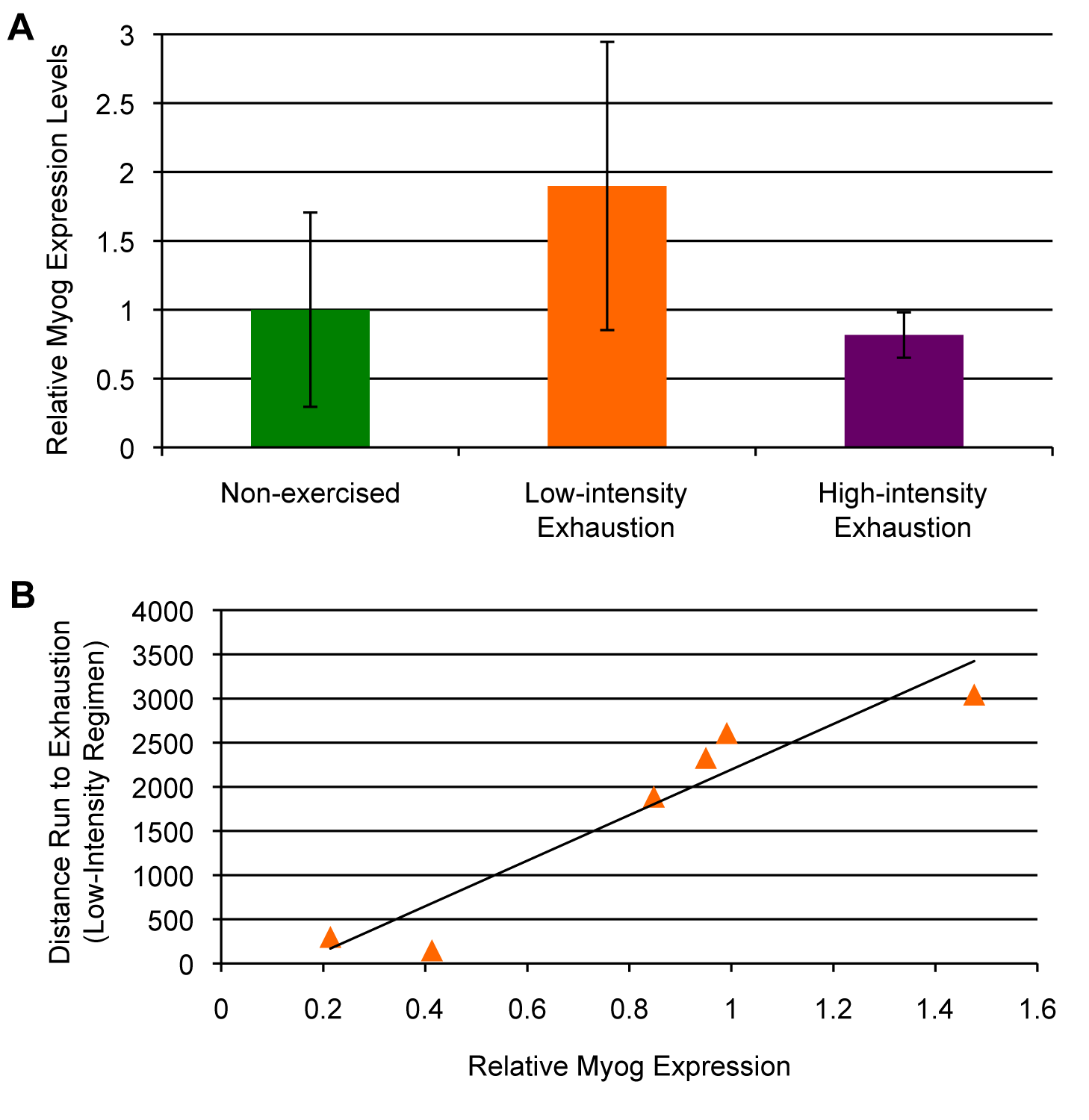

Supplement: Figure S4 — Myog expression increases in response to low-intensity exercise in wild type mice. (A) After running to exhaustion under the low-intensity regimen, wild-type mice exhibited a strong trend toward increased Myog transcript expression, but not after high-intensity running to exhaustion. (B) A directly proportionate relationship exists in wild-type mice between Myog transcript expression and distance run to exhaustion. Mice running greater distances possess increased Myog levels (R2 = 0.905 and Pearson correlation P = 0.013). Sedentary, n = 6; Low-intensity exhaustion, n = 6; High-intensity exhaustion, n = 6. Green bar indicates Myog expression values for sedentary group; orange bar indicates Myog expression values for sedentary group Myog-deleted values. Error bars represent one standard deviation. (0.13 MB TIF) [file pone.0013535.s004.tif]

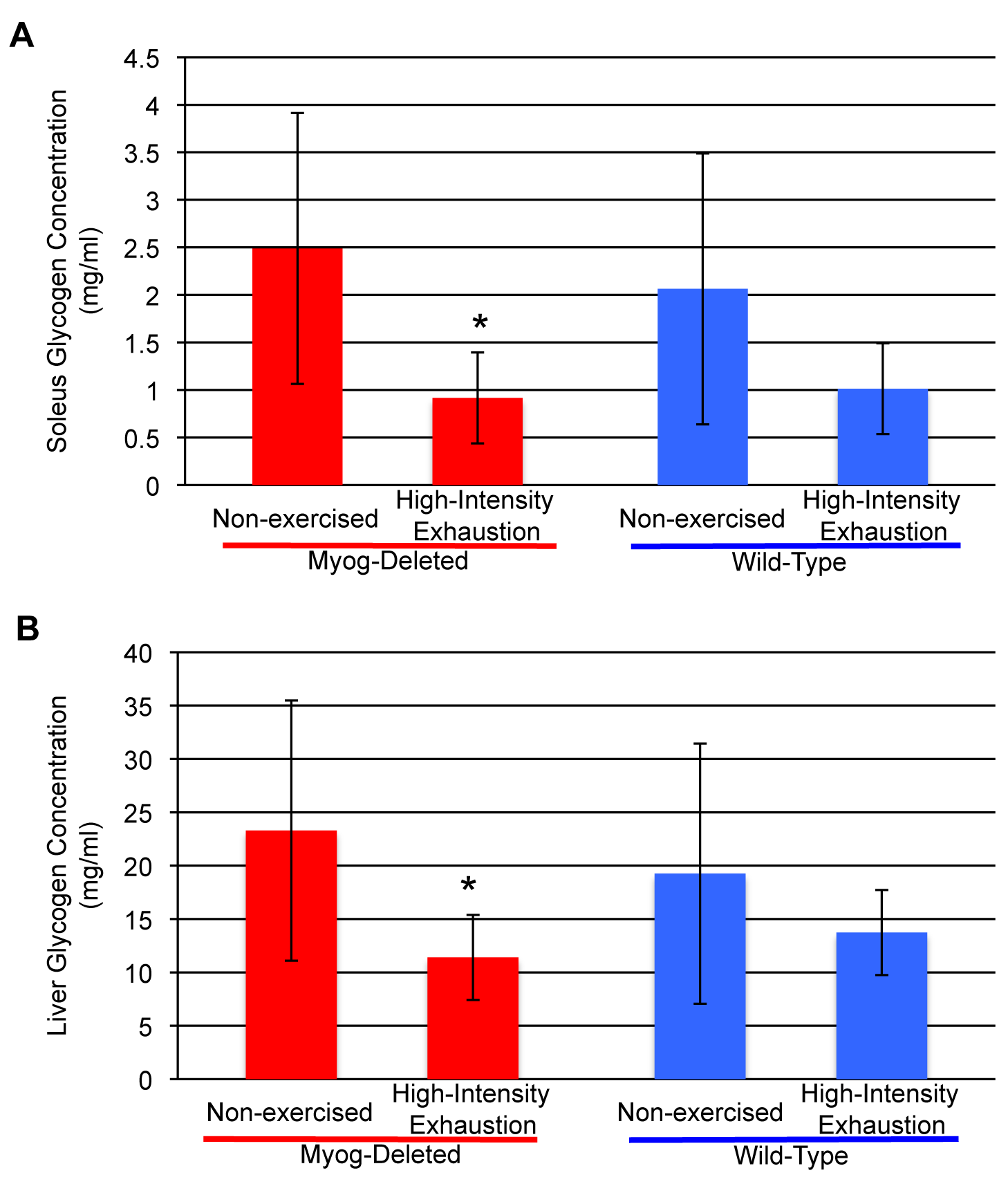

Supplement: Figure S5 — Soleus and liver glycogen concentration. Soleus (A) and liver (B) glycogen concentrations were significantly depleted in Myog-deleted mice (63% and 51%, respectively), but decreases in wild-type soleus and liver (51% and 21%, respectively) were less dramatic and not statistically significant. Myog-deleted, n = 6; wild-type, n = 6. Blue bars indicate wild-type control values; red bars indicate Myog-deleted values. Error bars represent one standard deviation. *P<0.05. (0.17 MB TIF) [file pone.0013535.s005.tif]

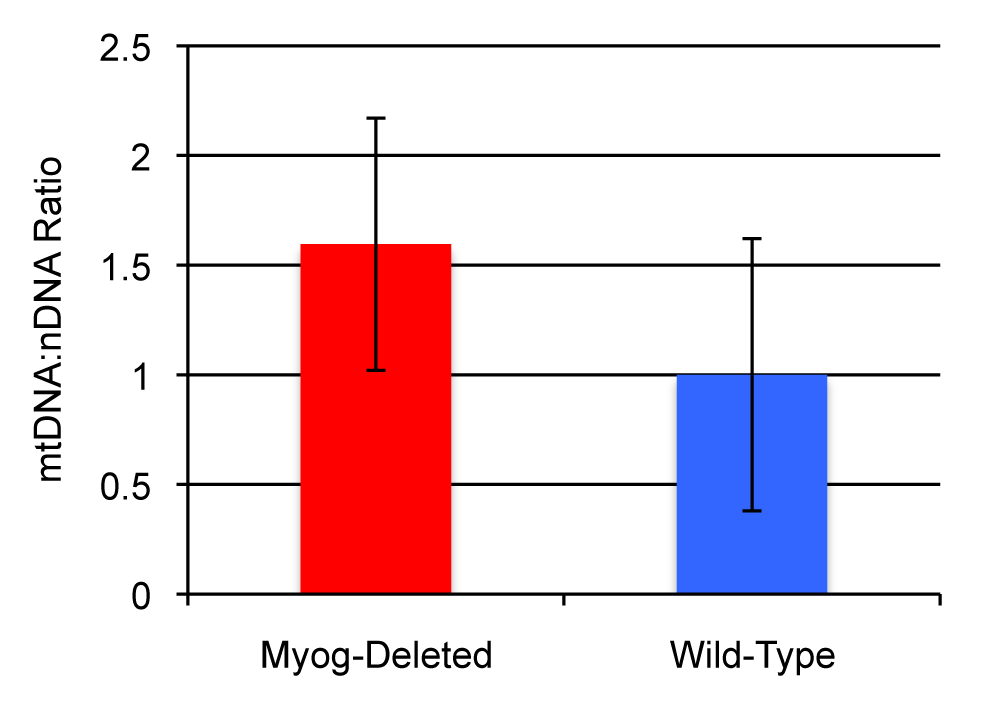

Supplement: Figure S6 — Mitochondrial abundance in gastrocnemius muscle of wild-type and Myog-deleted mice. Myog-deleted mice exhibited a strong trend toward an increased ratio of mitochondrial DNA to genomic DNA over the wild-type group (Myog-deleted, n = 6; wild-type, n = 6). Blue bars indicate wild-type control values; red bars indicate Myog-deleted values. Error bars represent one standard deviation. (0.06 MB TIF) [file pone.0013535.s006.tif]

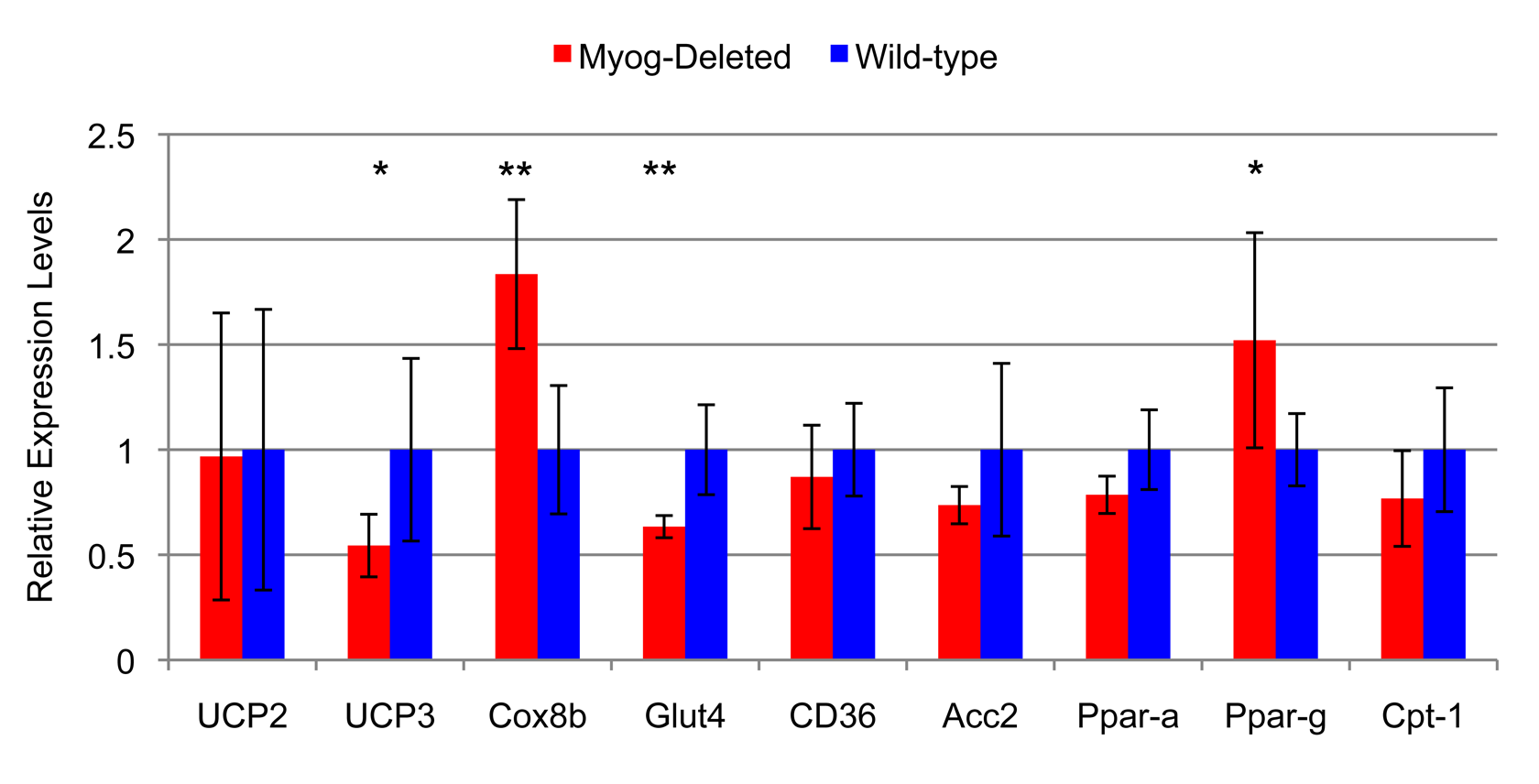

Supplement: Figure S7 — Myog-deleted mice exhibit normal expression of fatty-acid metabolism, mitochondrial function, and GLUT4 genes in gastrocnemius muscle. Taqman RT-qPCR of selected genes that play a role in metabolism include: Acc2 (acetyl-coenzyme A carboxylase beta, biosynthesis of fatty acids), CD36 (cluster of differentiation 36, fatty acid and glucose metabolism), Cox8b (cytochrome C oxidase subunit 8b, mitochondrial electron transport chain, oxidative ATP production), Cpt-1 (carnitine palmitoyltransferase I, transport of long chain fatty acids across mitochondrial membrane), Glut4 (glucose transporter type 4, glucose uptake), Ppar-a (peroxisome proliferator-activated receptor alpha, nuclear receptor, regulation of fatty acid storage and glucose metabolism), Ppar-g (peroxisome proliferator-activated receptor gamma, nuclear receptor, regulation of fatty acid storage and glucose metabolism), UCP2 (mitochondrial uncoupling protein 2, oxidative phosphorylation), and UCP3 (mitochondrial uncoupling protein 3, oxidative phosphorylation). Of the oxidative metabolism genes tested, the expression of Cox8b and Ppar-g was determined to be significantly increased in gastrocnemius muscle of Myog-deleted mice, (84% and 52%, respectively). UCP3 and Glut4 expression levels were significantly decreased (46% and 37%, respectively). Blue bars indicate wild-type control values; red bars indicate Myog-deleted values. (Myog-deleted, n = 6; wild-type, n = 6). Error bars represent one standard deviation. *p<0.05, **p<0.01 (0.14 MB TIF) [file pone.0013535.s007.tif]

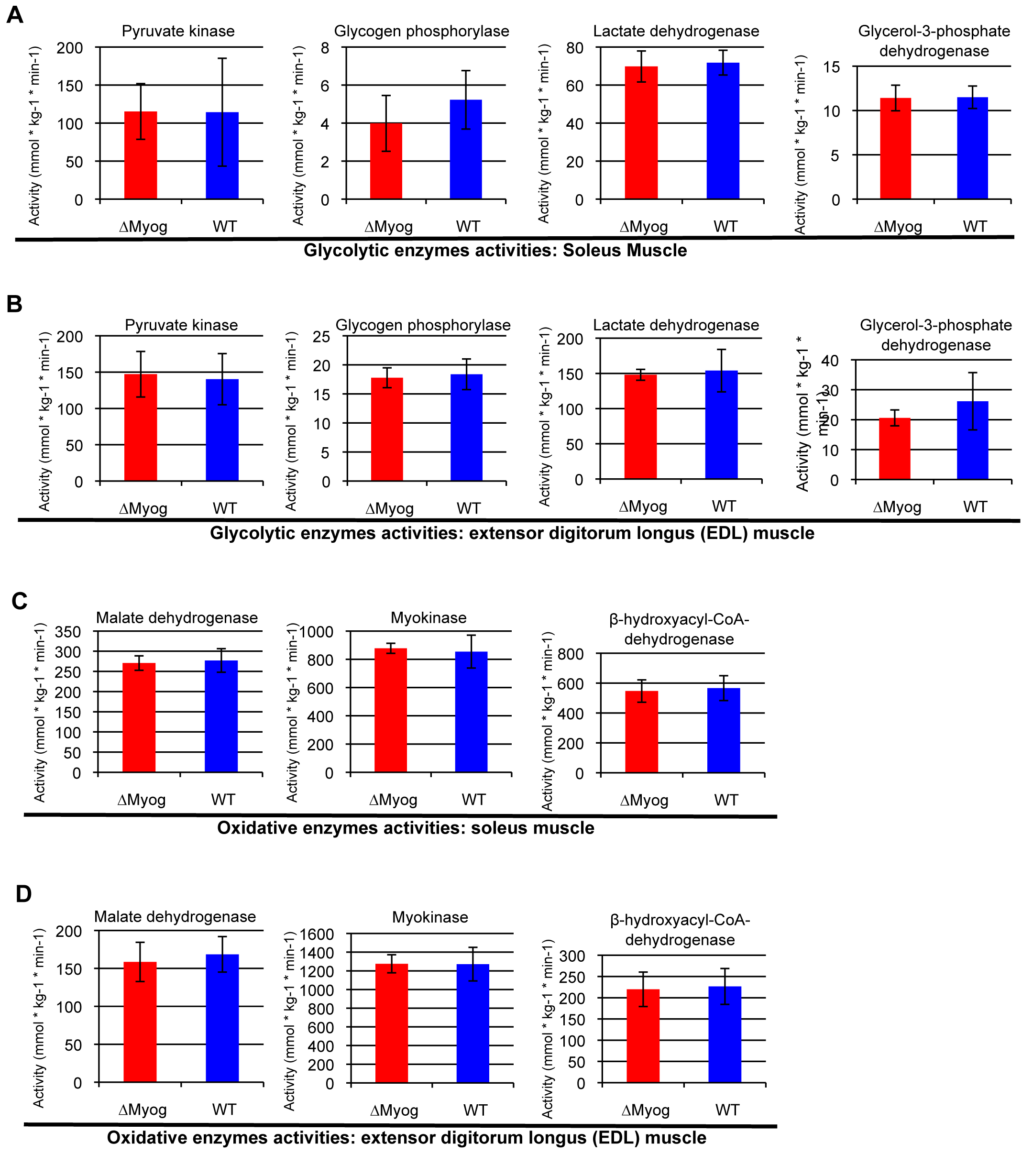

Supplement: Figure S8 — Myog-deleted mice exhibit normal oxidative and glycolytic metabolic enzyme activities in the soleus and extensor digitorum longus (EDL) muscles. (A) Glycolytic enzyme activities in the soleus muscle. (B) Glycolytic enzyme activities in the EDL muscle. (C) Oxidative enzyme activities in the soleus muscle. (D) Oxidative enzyme activities in the EDL muscle. Blue bars indicate wild-type control values; red bars indicate Myog-deleted values. (Myog-deleted, n = 4; wild-type, n = 4). Error bars represent one standard deviation. (0.38 MB TIF) [file pone.0013535.s008.tif]

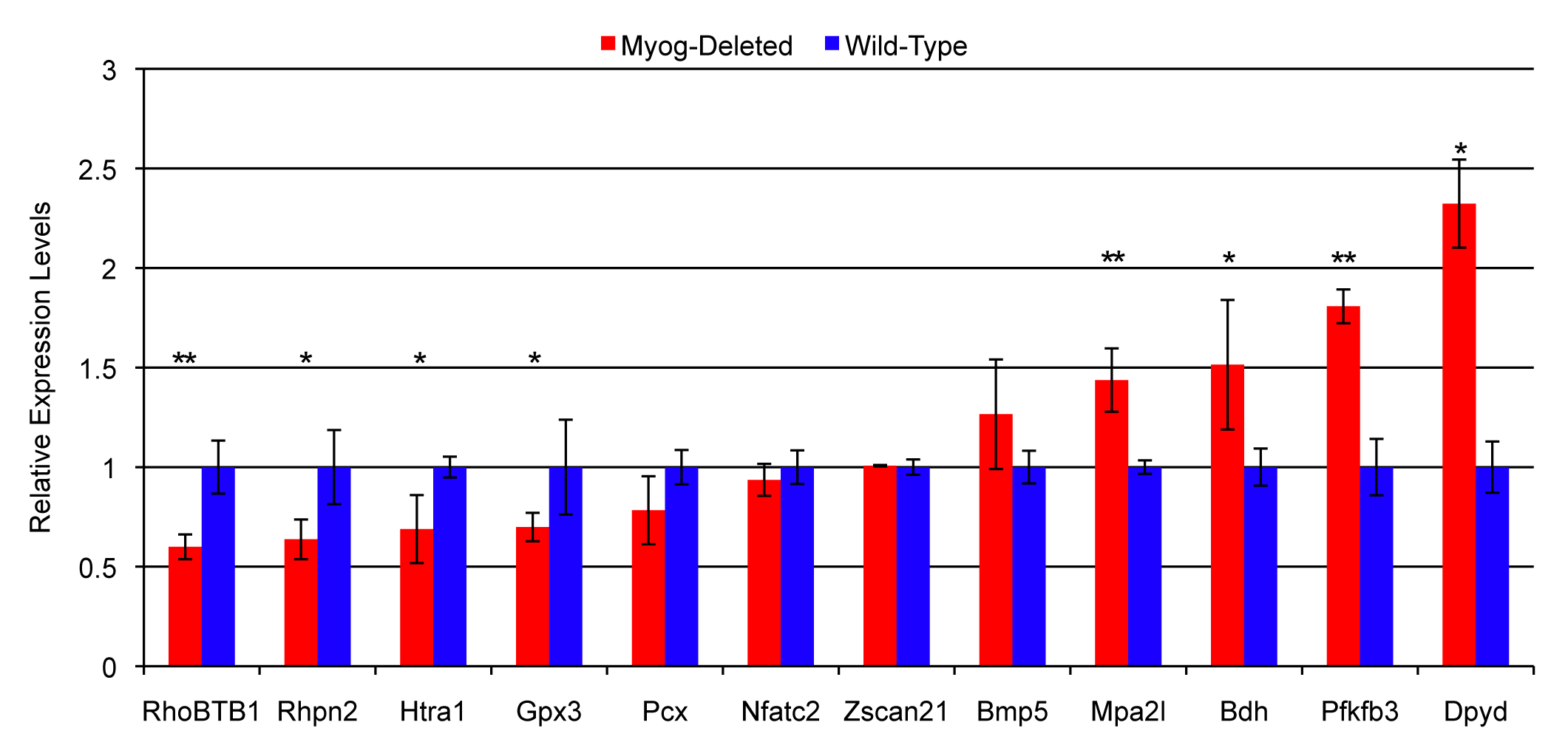

Supplement: Figure S9 — Myogenin regulates genes involved in metabolism and signal-transduction during adult life. Taqman RT-qPCR validation was performed on selected differentially expressed genes in adult Myog-deleted gastrocnemius muscle. Genes: RhoBTB1 (rho-related BTB domain containing 1, rho GTPase), Rhpn2 (rhophilin, rho GTPase binding protein), Htra1 (serine peptidase), Gpx3 (glutathione peroxidase 3), Pcx (pyruvate carboxylase), Nfatc2 (nuclear factor of activated T-cells 2), Zscan21 (zinc finger and SCAN domain containing 21), Bmp5 (bone morphogenetic protein 5), Mpa2l (macrophage activation 2 like), Bdh (3-hydroxybutyrate dehydrogenase), Pfkfb3 (phosphofructokinase/fructose-bisphosphatase 3), and Dpyd (dihydropyrimidine dehydrogenase). Blue bars indicate wild-type control values; red bars indicate Myog-deleted values. Error bars represent one standard deviation. *p<0.05, **p<0.01. (0.13 MB TIF) [file pone.0013535.s009.tif]
