# Supplementary material for: Myogenin Regulates Exercise Capacity and Skeletal Muscle Metabolism in the Adult Mouse
Source: PLoS One. 2010 Oct 22;5(10):e13535. doi: 10.1371/journal.pone.0013535 (PMC2962629; doi:10.1371/journal.pone.0013535)
Supplement: Table S1 — Gene expression profiling of Myog-deleted mice reveals differentially expressed genes involved in signal transduction and metabolism. (0.09 MB DOC) [file pone.0013535.s010.doc]

| **Probe Set ID** | **Fold change (DEL vs WT)** | **Regulation (DEL vs WT)** | **Unigene(Avadis)** | **Gene Symbol** | **Gene Title** | **Entrez Gene** |
| --- | --- | --- | --- | --- | --- | --- |
| 1418431_at | 1.4082962 | down | Mm.223744 | Kif5b | kinesin family member 5B | 16573 |
| 1438852_x_at | 1.4085702 | down | Mm.4933 | Mcm6 | minichromosome maintenance deficient 6 (MIS5 homolog, S. pombe) (S. cerevisiae) | 17219 |
| 1420514_at | 1.4119617 | down | Mm.441557 |  |  |  |
| 1459632_at | 1.4138387 | down |  |  |  |  |
| 1433634_at | 1.4195982 | down | Mm.334918 | Irf2bp2 | interferon regulatory factor 2 binding protein 2 | 270110 |
| 1422730_at | 1.4233496 | down | Mm.10987 | Limd1 | LIM domains containing 1 | 29806 |
| 1417083_at | 1.4310126 | down | Mm.20188 | Sec61b | Sec61 beta subunit | 66212 |
| 1428170_at | 1.4380435 | down | Mm.32254 | Zfp180 | zinc finger protein 180 | 210135 |
| 1429656_at | 1.4403652 | down | Mm.26659 | Rhobtb1 | Rho-related BTB domain containing 1 | 69288 |
| 1427426_at | 1.452653 | down | Mm.336519 | Kcnq5 | potassium voltage-gated channel, subfamily Q, member 5 | 226922 |
| 1443630_at | 1.4538457 | down |  |  |  |  |
| 1450420_at | 1.4641168 | down | Mm.42135 | LOC100045442 /// Stag1 | similar to Stromal antigen 1 /// stromal antigen 1 | 100045442 /// 20842 |
| 1431708_a_at | 1.4743853 | down | Mm.423551 | Tia1 | cytotoxic granule-associated RNA binding protein 1 | 21841 |
| 1457458_at | 1.4814531 | down | Mm.333594 | Zc3h4 | zinc finger CCCH-type containing 4 | 330474 |
| 1458551_at | 1.498179 | down |  | AY512938 | cDNA sequence AY512938 | 791388 |
| 1438251_x_at | 1.4985213 | down | Mm.30156 | Htra1 | HtrA serine peptidase 1 | 56213 |
| 1444057_at | 1.5008023 | down | Mm.446146 |  |  |  |
| 1416383_a_at | 1.507533 | down | Mm.1845 | Pcx | pyruvate carboxylase | 18563 |
| 1455364_a_at | 1.5083363 | down | Mm.279839 | EG640050 /// Rps7 | predicted gene, EG640050 /// ribosomal protein S7 | 20115 /// 640050 |
| 1423854_a_at | 1.5361482 | down | Mm.293316 | Rasl11b | RAS-like, family 11, member B | 68939 |
| 1449732_at | 1.5632496 | down | Mm.2760 | Zscan21 | zinc finger and SCAN domain containing 21 | 22697 |
| 1449106_at | 1.5641023 | down | Mm.200916 | Gpx3 | glutathione peroxidase 3 | 14778 |
| 1422731_at | 1.595016 | down | Mm.10987 | Limd1 | LIM domains containing 1 | 29806 |
| 1416749_at | 1.6138359 | down | Mm.30156 | Htra1 | HtrA serine peptidase 1 | 56213 |
| 1419391_at | 1.7290214 | down | Mm.16528 | Myog | myogenin | 17928 |
| 1434628_a_at | 1.7672355 | down | Mm.286600 | Rhpn2 | rhophilin, Rho GTPase binding protein 2 | 52428 |
| 1429206_at | 1.8050308 | down | Mm.26659 | Rhobtb1 | Rho-related BTB domain containing 1 | 69288 |
| 1422128_at | 2.224399 | down | Mm.289810 | Rpl14 | ribosomal protein L14 | 67115 |
| 1460567_at | 2.3582141 | down | Mm.32081 | Rfx7 | regulatory factor X, 7 | 319758 |
| 1440068_at | 3.242296 | down |  |  |  |  |
| 1447927_at | 1.4303422 | up | Mm.458435 | EG634650 /// Gbp10 /// Mpa2l | predicted gene, EG634650 /// guanylate-binding protein 10 /// macrophage activation 2 like | 100702 /// 626578 /// 634650 |
| 1427722_at | 1.4324526 | up | Mm.269823 | Slc15a5 | solute carrier family 15, member 5 | 277898 |
| 1455851_at | 1.4348811 | up | Mm.473755 | Bmp5 | bone morphogenetic protein 5 | 12160 |
| 1419097_a_at | 1.4425579 | up | Mm.295284 | Stom | stomatin | 13830 |
| 1427384_at | 1.4685605 | up | Mm.122738 | Chd6 | chromodomain helicase DNA binding protein 6 | 71389 |
| 1421955_a_at | 1.4862177 | up | Mm.279923 | Nedd4 | neural precursor cell expressed, developmentally down-regulated 4 | 17999 |
| 1416432_at | 1.5562963 | up | Mm.19669 | Pfkfb3 | 6-phosphofructo-2-kinase/fructose-2,6-biphosphatase 3 | 170768 |
| 1438676_at | 1.7935469 | up | Mm.275893 | Mpa2l | macrophage activation 2 like | 100702 |
| 1427946_s_at | 1.931305 | up | Mm.27907 | Dpyd | dihydropyrimidine dehydrogenase | 99586 |
| 1426959_at | 1.9909414 | up | Mm.293470 | Bdh1 | 3-hydroxybutyrate dehydrogenase, type 1 | 71911 |
| 1421244_at | 79.52962 | up | Mm.463262 | Esr1 | estrogen receptor 1 (alpha) | 13982 |
